# Tandem gene arrays in *Trypanosoma brucei*: Comparative phylogenomic analysis of duplicate sequence variation

**DOI:** 10.1186/1471-2148-7-54

**Published:** 2007-04-04

**Authors:** Andrew P Jackson

**Affiliations:** 1 Wellcome Trust Sanger Institute, Wellcome Trust Genome Campus, Hinxton, Cambridgeshire, CB10 1SA, UK

## Abstract

**Background:**

The genome sequence of the protistan parasite *Trypanosoma brucei *contains many tandem gene arrays. Gene duplicates are created through tandem duplication and are expressed through polycistronic transcription, suggesting that the primary purpose of long, tandem arrays is to increase gene dosage in an environment where individual gene promoters are absent. This report presents the first account of the tandem gene arrays in the *T. brucei *genome, employing several related genome sequences to establish how variation is created and removed.

**Results:**

A systematic survey of tandem gene arrays showed that substantial sequence variation existed across the genome; variation from different regions of an array often produced inconsistent phylogenetic affinities. Phylogenetic relationships of gene duplicates were consistent with concerted evolution being a widespread homogenising force. However, tandem duplicates were not usually identical; therefore, any homogenising effect was coincident with divergence among duplicates. Allelic gene conversion was detected using various criteria and was apparently able to both remove and introduce sequence variation. Tandem arrays containing structural heterogeneity demonstrated how sequence homogenisation and differentiation can occur within a single locus.

**Conclusion:**

The use of multiple genome sequences in a comparative analysis of tandem gene arrays identified substantial sequence variation among gene duplicates. The distribution of sequence variation is determined by a dynamic balance of conservative and innovative evolutionary forces. Gene trees from various species showed that intraspecific duplicates evolve in concert, perhaps through frequent gene conversion, although this does not prevent sequence divergence, especially where structural heterogeneity physically separates a duplicate from its neighbours. In describing dynamics of sequence variation that have consequences beyond gene dosage, this survey provides a basis for uncovering the hidden functionality within tandem gene arrays in trypanosomatids.

## Background

Evolutionary biology has begun to utilise the abundance of genome sequence data, applying a comparative approach to the evolutionary dynamics of genome structure and sequence [1-3]. Within this approach genomic position is a novel source of contextual information, and an additional criterion for assessing homology, independent of gene sequences. The debate has recently concentrated on the relative contributions of evolutionary processes affecting sequence divergence, such as gene conversion, positive and negative selection and concerted evolution, and processes affecting gene quantity and order [4,5]. This study concerns the evolution of tandem gene arrays in the kinetoplastid parasite *Trypanosoma brucei*, the cause of sleeping sickness in humans and various diseases in animals, for which a genome sequence was recently completed [6]. A comparative method, using genome sequence from multiple trypanosomatid species, was applied to tandem-duplicated genes throughout the *T. brucei *genome to test for various evolutionary forces. The results reveal the variety of dynamics among tandem arrays and clarify the contribution of concerted evolution and gene conversion in regulating sequence diversity among repetitive genes.

Long before the appearance of genome sequences, tandem gene arrays were known to affect genome structure and gene function. Ribosomal rDNA is commonly tandemly-duplicated across the taxonomic spectrum, and is typically thought to be homogenised as a result [7]. In contrast, metazoan *hox *genes are also tandemly arrayed, but contain substantial variation that demonstrates the functional consequences of the array organisation [8]. Tandem arrays are thought to evolve through a process of replication slippage during DNA synthesis or unequal crossing over during meiosis [9], a phenomenon conceptually consistent with the rapid evolution of mini-satellites and other highly repetitive regions in many genomes through mis-alignment [10]. These processes often result in contiguous gene copies evolving in concert toward a consensus sequence, such that duplicate sequences within tandem arrays may fail to diverge as expected under a neutral model [9]. In these circumstances gene duplicates within a species look more alike than orthologous loci in different species, which is indicative of concerted evolution [11]. Concerted evolution of duplicated genes was first recognised among the rDNA of *Xenopus *[12] and subsequently shown to be widespread among the globins of primates [13,14] and heat-shock proteins in *Drosophila *[15]. Furthermore, unequal crossing-over (UCO) and gene conversion (GC) were suggested as mechanisms for this homogenisation within arrays, although their relative contributions were unknown [16], and remain so.

UCO results from mis-alignment of repetitive DNA and occurs between sister chromatids during mitosis and between homologous chromosomes during meiosis [9]; it principally affects gene numbers and can cause both the addition and subtraction of repeat units, as shown in Additional file 1. Consequently, tandem arrays affected by UCO can show intraspecific copy number variation; but while copy number can vary, gene sequences are relatively invariant because UCO adds or subtracts whole repeat units. UCO was implicated in the formal description of concerted evolution among tandem α-globins in primates [18] and is also known to occur in *trans*, for example, among the five unlinked rDNA loci in humans [19]. The removal of variation by UCO was demonstrated in Streptococci where the functional polymorphism of rDNA tandem copies, which facilitates antibiotic resistance, was abolished when diversifying selection was removed from the bacteria [17].

GC describes the reciprocal or directed exchange of material between alleles on homologous chromosomes or between gene duplicates; these may be at the same locus (allelic gene conversion, AGC) or at different loci (ectopic gene conversion, EGC). Reciprocal exchange between alleles on homologous chromosomes is commonly understood as 'recombination', while non-reciprocal exchanges within or between loci are termed 'biased gene conversion'. These processes are also illustrated in Additional file 1. This study primarily concerns exchanges among gene duplicates, within or between loci, regardless of their reciprocity (although most are biased events). First observed in fungi as a mechanism for homogenisation of alleles [20], GC can homogenise sequences, for instance among mammalian [21] and nematode [22] heat-shock proteins, but also promote polymorphism, for example, in the diversification of both immunoglobulin [23,24] and multi-histocompatibility complex alleles [25,26].

Detecting the action of concerted evolution is essentially about identifying similarities where none are expected; previously, concerted evolution has been identified by eye from obvious, anomalous similarities [27-30]. Genomic position is central to showing that a lack of divergence was not caused by purifying selection or recent gene duplications. The contextual information provided by genome sequences show that, first, there is a substantial excess of mutations between duplicates in different species, compared to duplicates in the same genome, and second, because gene duplicates display conserved synteny with surrounding loci, they were established long ago. Quantitative methods for the treatment of GC and UCO have been developed in the last three decades (for a review see [31]) and were applied in this study under four criteria:

• *Cladistic criterion*: homologous genes from several organisms are arranged in a phylogenetic tree to estimate their cladistic relationships; concerted evolution is inferred where homologous genes cluster by species, rather than by locus, i.e., paralogs appear more similar than expected given that their closest relatives are found in other genomes.

• *Phylogenetic criterion*: a 'sliding window' analysis [32,33] is applied to a multiple alignment of homologous genes, with phylogenetic trees estimated for each window. A significant departure in phylogenetic signal between windows identifies two regions with different evolutionary histories, i.e., a recombination 'breakpoint'.

• *Distribution criterion *[34]: the distribution of silent polymorphisms along a sequence alignment is scored for areas where the distribution is 'condensed', i.e., matches between two otherwise discordant sequences. Where the length of a corresponding region is significantly larger than expected after permutation, a gene conversion event is inferred.

• *Compatibility criterion *[35]: two homologous genes are compared on a site-by-site basis, breakpoints are inferred where neighbouring, informative sites have incompatible phylogenetic signals.

The importance of concerted evolution has been demonstrated through whole genome comparisons of gene duplicates, across diverse taxa [36-39], but in most detail for yeast [31,40,41]. Most of these studies have compared duplicated sequences within genomes, but have not provided a comparative context for sequence evolution. In this study, tandem gene arrays were chosen because they offer a structural paradigm to infer evolutionary events, and one peculiar to trypanosomatids since they typically lack individual gene promoters and transcribe polycistronically [42,43,6]. Therefore, the argument that tandem duplication serves to increase gene dosage in the absence of promoters [44] could be assessed. A comparative approach was also specifically chosen, in which variation among *T. brucei *duplicate sequences was analysed in relation to homologous sequences in five other kinetoplastids, ranging in relation from a subspecies (*T. b. gambiense*) to another genus (*L. major*). This provided the historical perspective necessary to identify the origins and causes of sequence variation. Hence, the specific aims were to (i) catalogue all tandem gene arrays in the genome sequence, establish their phylogenetic distribution in other trypanosomatid species and quantify the variation found among the duplicate sequences of each; (ii) assess the relative contributions of concerted evolution, UCO and GC, through the application of multiple tests; and (iii) use this information to characterise the causes of variation (or lack of) in each array.

## Results

After surveying the *Trypanosoma brucei *genome sequence, 47 tandem arrays with at least four gene copies were identified and characterised. This is unlikely to be an exhaustive list of tandem-duplicated loci in the *T. brucei *genome since some lengthy tandem arrays may be collapsed to tandem pairs or triplets if gene number could not be resolved during genome assembly. A full list of tandem-duplicates, regardless of copy number, is available from the author. Duplicate sequences were combined with homoeologous sequences (i.e., homologous sequences occurring at the same genomic position) from five other trypanosomatid species and several criteria were applied to the multiple alignments to identify the molecular signatures of concerted evolution and gene conversion. Each of the methods scrutinised multiple alignments for unexpected similarity between otherwise dissimilar gene sequences, which could not be explained through occasional conservation or derivation. The evidence suggested that tandem arrays display a variety of evolutionary dynamics and often contain substantial sequence variation; among them examples of conserved arrays, evolving in copy number through unequal crossing-over, moderately variable duplicates evolving in concert, those affected by allelic gene conversion, and also structurally segregated arrays, with different duplicates exposed to distinct evolutionary forces.

### Variation within tandem arrays

Values for *D*_*n *_and *D*_*s *_in Table 1 show that for many cases there was substantial variation among the duplicate genes within tandem arrays. Indeed, in a few cases *D*_*s *_exceeded 1 (6 cases), probably due to highly divergent copies in these arrays that showed little or no sequence homology over part of the alignment. Typically, tandem arrays showed moderate variation among copies, where *D*_*s *_always exceeded *D*_*n*_, but *D*_*n *_was non-zero (31 cases). However, a sizable minority showed no variation at all (10 cases).

**Table 1:** Tandem gene arrays in *Trypanosoma brucei*: phylogenetic distributions, sequence variation and results of SH tests.

Identifier	Description	Distribution*:						Average sequence divergence^†^:		Cladistic criterion^#^:								
		Tb	Tbg	Tco	Tv	Tc	Lm	*D*_*s*_	*D*_*n*_	Bootstrap	Posterior probability	Log Likelihood:						
												Optimal	Orthologous	Concerted				
														Δ-lnL	p		Δ-lnL	p
3	α and β-tubulin	X	X	X	X	X		0.0000	0.0000	500	1.00	-	-	-	-	-	-	-
4	histone H3	X	X	X				0.0000	0.0000	500	1.00	-	-	-	-	-	-	-
6	Tb927.1.4540	X	X	X	X			0.2068	0.1227	423	1.00	-2448.9	-3532.7	-1083.8	**0.0001**	-2448.9	0	0.759
9	65 kDa invariant surface glycoprotein	X	X					0.1545	0.0763	-	-	-	-	-	-	-	-	-
12	Tb927.2.5290	X	X					0.1559	0.0775	-	-	-	-	-	-	-	-	-
13	Tb927.3.2550	X	X					0.1557	0.0859	-	-	-	-	-	-	-	-	-
17	Tb927.3.4070	X	X	X	X	X	X	0.1680	0.0664	499	1.00	-11194.3	-14196.6	-3002.3	**0.0001**	-11204.7	-10.4	0.448
18	73 kDa paraflagellar rod protein	X	X	X	X	X	X	0.0010	0.0000	500	0.55	-5510.7	-7529.3	-2018.6	**0.0001**	-5510.7	0	0.506
20	Tb927.3.5690	X						0.4137	0.2155	-	-	-	-	-	-	-	-	-
24	serine/threonine-protein phosphatase PP1	X	X	X	X	X	X	1.5094	0.2409	500	1.00	-5352.4	-6392	-1039.6	**0.0001**	-6068.6	-716.2	**0.0001**
26	amino acid transporter	X	X	X	X			0.3427	0.1226	493	1.00	-6465.23	-6892.64	-427.41	**0.0001**	-6465.23	0	0.749
28	receptor-type adenylate cyclase GRESAG 4	X	X					0.1041	0.0647	-	-	-	-	-	-	-	-	-
29	amino acid transporter 10	X	X					0.5819	0.2133	-	-	-	-	-	-	-	-	-
30	UDP-GlcNAc-dependent glycosyltransferase	X	X	X				0.0425	0.0286	500	1.00	-4356.7	-6723.1	-2366.4	**0.0001**	-4366.8	-10.1	0.423
32	75 kDa invariant surface glycoprotein	X	X					1.1899	0.4529	-	-	-	-	-	-	-	-	-
39	histone H4	X	X	X	X	X		0.0000	0.0000	500	0.95	-	-	-	-	-	-	-
42	receptor-type adenylate cyclase GRESAG 4	X						0.0262	0.0135	-	-	-	-	-	-	-	-	-
43	cysteine peptidase	X	X	X	X	X		0.0000	0.0000	499	1.00	-	-	-	-	-	-	-
44	Tb927.6.1300	X						0.0220	0.0043	-	-	-	-	-	-	-	-	-
47	S-adenosylmethionine synthetase	X	X	X				0.0000	0.0000	500	1.00	-	-	-	-	-	-	-
55	retrotransposon hot spot protein 7 (RHS7)	X	X					0.0658	0.0647	-	-	-	-	-	-	-	-	-
57	histone H2A	X	X	X	X	X		0.0000	0.0000	500	0.98	-	-	-	-	-	-	-
61	Tb927.7.5930	X	X					0.3745	0.1181	-	-	-	-	-	-	-	-	-
62	receptor-type adenylate cyclase GRESAG 4	X	X					0.0490	0.0337	-	-	-	-	-	-	-	-	-
62a	Tb927.7.6110	X	X	X				0.8373	0.2748	493	na	-3876.7	-3991.1	-114.4	**0.0001**	-3876.7	0	0.501
64a	nucleolar RNA-binding protein	X	X	X	X	X	X	0.7310	0.5628	412	1.00	-2830.7	-2822.5	8.2	0.482	-3175.9	-345.2	**0.0001**
67	major surface protease gp63	X	X	X				0.0083	0.0090	500	1.00	-4693	-6266	-1573	**0.0001**	-4677.2	15.8	0.391
72	amino acid transporter	X	X	X	X	X		0.0127	0.0062	500	na	-4798.5	-6752.3	-1953.8	**0.0001**	-4872.6	-74.1	0.11
73	PFR2 69 kDa paraflagellar rod protein	X	X	X	X	X	X	0.0000	0.0000	500	1.00	-	-	-	-	-	-	-
75	Tb927.8.6700	X	X	X	X	X	X	0.7726	0.3661	500	1.00	-15754.1	-18536.1	-2782	**0.0001**	-15812.6	-58.5	0.194
80	amino acid transporter	X	X	X				0.7609	0.1607	497	1.00	-5761	-6677.5	-916.5	**0.0001**	-6844.1	-1083.1	**0.0001**
80a	receptor-type adenylate cyclase GRESAG 4	X	X	X				0.0624	0.0361	480	1.00	-19488.9	-25099.5	-5610.6	**0.0001**	-19498.2	-9.3	0.468
82	fatty acyl CoA syntetase	X	X	X	X	X	X	1.2045	0.3632	na	na	-19561.6	-19987.6	-426	**0.001**	-21771.5	-2209.9	**0.0001**
85	Tb09.v1.0470	X	X					0.5568	0.2667	-	-	-	-	-	-	-	-	-
87a	Tb09.211.1000	X	X	X	X			0.1673	0.0786	500	1.00	-7085	-8471	-1386	**0.0001**	-7092.9	-7.9	0.42
89	glycerol kinase	X	X	X	X	X	X	0.0050	0.0012	374	1.00	-3369.3	-3883.5	-514.2	**0.0001**	-3379.3	-10	0.425
90	ADP-ribosylation factor	X	X	X	X	X		0.0119	0.0012	500	1.00	-1805.1	-2709.1	-904	**0.0001**	-1805.1	0	0.736
93	Bloodstream Alanine Rich Protein (BARP)	X	X	X				0.5033	0.2534	500	na	-4060.3	-4639.6	-579.3	**0.0001**	-4063.4	-3.1	0.457
103	Tb10.70.0040	X	X	X	X	X	X	0.4821	0.2926	500	1.00	-9380.5	-10906.8	-1526.3	**0.0001**	-9380.5	0	0.773
105a	expression site-associated gene (ESAG) protein	X	X	X	X			0.5109	0.4099	500	1.00	-8318.4	-10357.7	-2039.3	**0.0001**	-8384.3	-65.9	0.104
106	procyclin-associated gene	X	X	X				1.6527	0.9281	346	0.91	-8085.9	-8130.6	-44.7	**0.014**	-8221.1	-135.2	**0.0001**
107	histone H2B	X	X	X	X	X		0.0000	0.0000	499	0.99	-	-	-	-	-	-	-
109c	Tb10.389.0830	X	X	X	X	X		1.8231	0.6691	na	na	-6267.9	-7327.1	-1059.2	**0.0001**	-6387	-119.1	**0.017**
112	DNA polymerase kappa	X	X	X	X	X	X	0.2129	0.0892	500	1.00	-5710	-6440.8	-730.8	**0.0001**	-5710	0	0.511
113	cation transporter	X	X	X	X	X	X	1.6028	0.2454	na	1.00	-4974.1	-7292.3	-2318.2	**0.0001**	-4982.4	-8.3	0.444
117	calmodulin	X	X	X	X	X	X	0.0056	0.0000	414	1.00	-	-	-	-	-	-	-
x1	ribonucleoside-diphosphate reductase small chain	X	X					0.0101	0.0126	-	-	-	-	-	-	-	-	-

* Codes for species names: Tb: *T. brucei*; Tbg: *T. b. gambiense*; Tco: *T. congolense*; Tv: *T. vivax*; Tc: *T. cruzi*; Lm: *L. major*. Presence of a homologous locus within conserved gene order is shown by a cross X.

^† ^Sequence divergence is expressed using the rate of synonymous (D_s_) and non-synonymous (D_n_) substitutions per site.

^# ^Monophyly of array copies was assessed through non-parametric bootstraps of a ML phylogeny (500 replicates) and posterior probabilities in a Bayesian phylogeny ('na' denotes that monophyly was not recovered by the Bayesian tree). SH tests compared the likelihood of an optimal topology with 'orthologous' and 'concerted' scenarios; significant p-values are shown in bold and indicate that the scenario concerned was significantly less likely that the optimal topology. The cladistic criterion was only applied to arrays with widespread distributions.

Turning first to the invariant arrays, these included histones (Tb4, 39, 57, 107), tubulin (Tb3), cysteine peptidase (Tb43), glycerol kinase (Tb89), paraflagellar rod proteins (Tb18, 73) and S-adenosylmethionine synthetase (Tb47). The coding sequences of these arrays were identical, except for very occasional silent changes. The non-coding parts were typically identical also, although there was some variation in copy number of repetitive tracts, for instance in the 3' UTR of Tb4 (although the ability to precisely determine tract length is limited). Generally, the terminal UTRs of the arrays were identical to internal IGSs but curtailed; this shortening was assumed to reflect the proportion of the IGS properly assigned as UTR.

Among moderately variable cases, variation was typically continuous among copies and greater in coding regions. Notable examples of this included Tb93 (BARPs), where the corrected genetic distance between coding sequences (CDS) was 0.335 sub/site, while equivalent values for 5' and 3' UTRs were 0.073 and 0.038 respectively. In other arrays, such as Tb24 (S-T phosphatase) and Tb62a, the non-coding regions were invariant, while their associated CDS showed significant divergence. The only instances to show the opposite pattern, where non-coding regions were more variable than CDS were the histone arrays (Tb4, 39 and 107), albeit because the CDSs were invariant.

Where variation existed, the phylogenetic signal among coding sequences was typically inconsistent with 5' and 3' UTRs. Additional file 2 shows phylogenetic networks for three components of Tb55 (retrotransposon hot-spot protein). In frame A, copies 2–4 are robustly divergent from other duplicates, but this was not repeated with either 5' or 3' UTRs (frames C and D respectively). Not only are copies 2–4 dispersed in these latter networks, but nowhere do the UTRs show the same pattern at CDSs. Hence, these regions of the repeat unit showed different histories and evolutionary rates. Similarly, the relationships between Tb61 CDSs, shown in Additional file 3, are not reflected in the affinity of UTRs, of which there are three distinct and unrelated types. The exceptions to this inconsistency were those arrays showing discontinuous and mixed patterns of variation (see below).

**Figure 1:**
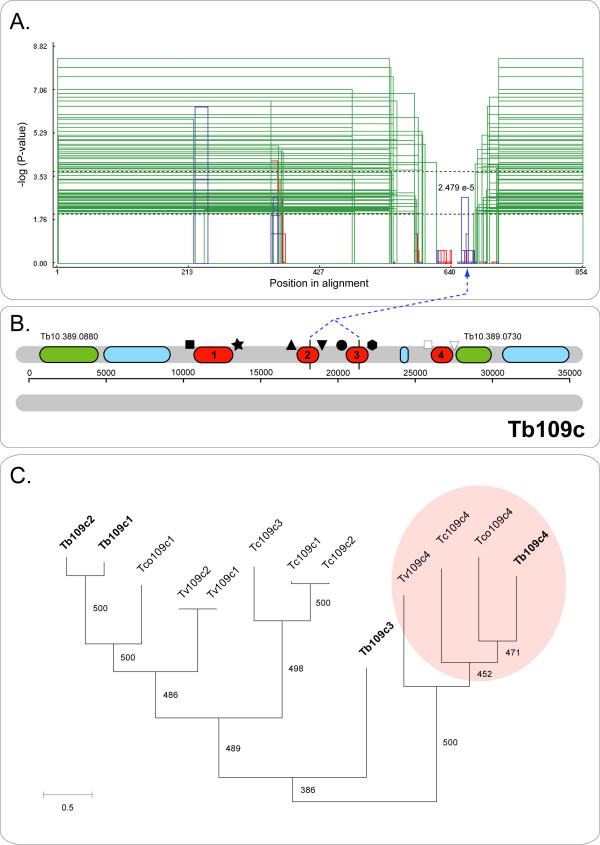
Mixed patterns of variation within array Tb109c (hypothetical protein, Tb10.389.0740). A. GENECONV output after analysis of an alignment of gene copies 1–3. Similarity was inferred from the non-random distribution of shared polymorphisms among any two sequences; the likelihood of each phylogenetic grouping is represented by coloured rectangles: copies 1 and 2 as closest relatives (green); 1 and 3 (red); 2 and 3 (blue). B. Diagram of the chromosomal position of Tb109c. DNA strands are shown in grey, scale is in base-pairs. The tandem array is coloured red, other annotated genes are green and hypothetical genes are coloured blue. The identity of UTRs around each gene duplicate is denoted by geometric symbols; identical UTRs possess the same symbol. C. Phylogenetic tree for Tb109c homologs from four species; orthology is retained by the divergent fourth duplicate, producing a distinct clade (shaded). Values are bootstrap proportions out of 500.

**Figure 2:**
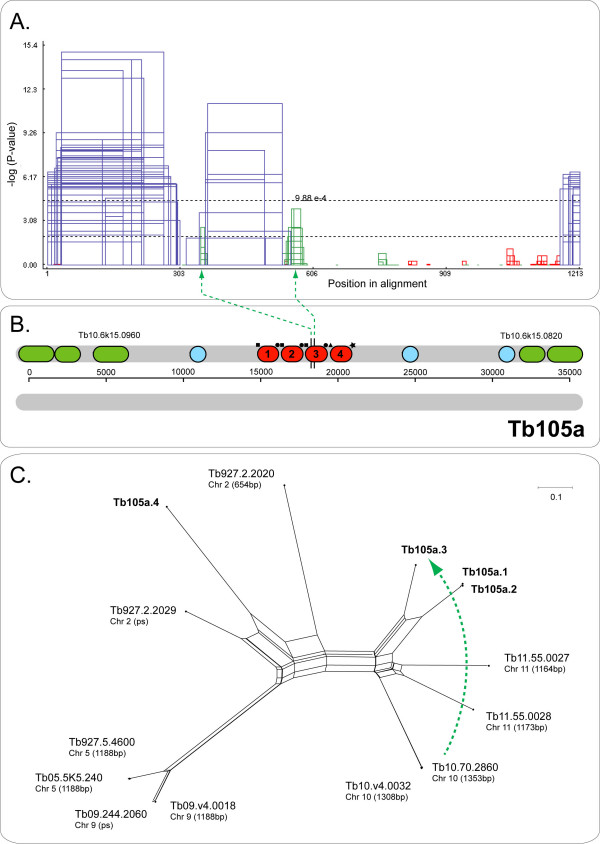
Ectopic gene conversion affecting array Tb105a (expression-site associated gene, Tb10.6k15.0910), identified by distribution criterion. A. GENECONV output, showing significant similarity between regions in an alignment of gene copies 2 and 3 and another gene at a locus in *trans *(Tb10.70.2860). Regions of significant similarity are represented by coloured rectangles, where colour refers to the two sequences concerned (blue: Tb105a.2 and Tb105a.3; red: Tb105a.2 and Tb10.70.2860; green: Tb105a.3 and Tb10.70.2860). Dashed lines denote significance thresholds. B. Diagram of the chromosomal position of Tb105a. Dashed green lines link Tb105a.3 with significant breakpoints in the alignment. C. Phylogenetic network of Tb105a gene copies and related loci throughout the genome, produced using the Neighbour-Net algorithm in Splitstree v4.0. The dashed green line shows the direction of the putative ectopic gene conversion.

Some tandem arrays containing highly variable duplicates were idiosyncratic. Figure 1 describes Tb109c, which contained four copies with extensive length differences that were genuinely divergent from one another. Other arrays with high average *D*_*s *_obtained this value due to discontinuous variation, that is, they all included an atypical duplicate that did not represent the patterns shown by other copies. Instances where divergence was significantly greater between some duplicates than others are referred to as having 'mixed dynamics'. Tb24 (see Additional file 4) included a divergent copy at its 3' end that lacked the C- and N-termini of other repeats. Tb32 (75 kDa invariant surface glycoprotein) included a divergent copy at its 5' end that differed from other duplicates through frequent indels. Tb113 (cation transporter) and Tb82 also possessed divergent copies at their 5' ends. In these cases, the divergent gene was the exception and other copies varied continuously. The divergent copies were always accompanied by radically remodelled IGSs, which failed to align with other non-coding repeats within the array. For instance, Tb105a is shown in Figure 2; its last copy had a distinctive C-terminus and both 5' and 3' UTRs bore no resemblance to others in the array. This often resulted in obvious structural change within the array, with the divergent copy being physically segregated from the remainder. Consequently, CDS and IGS relationships usually coincided for these cases.

In summary, duplicate genes within tandem arrays typically contained variation and not all elements of the array provided an identical phylogenetic signal. Some duplicates were physically segregated from the remaining array by divergent non-coding sequence; where this had occurred, these genes diverged substantially and variation was distributed discontinuously.

### Evidence for concerted evolution

A cladistic criterion was applied to each sequence alignment 'C', to infer concerted evolution, i.e., where paralogous sequences within a species looked more alike than their putative homoeologs in other species. It was trivial to confirm this for invariant arrays, since all copies were identical, but the likelihood of concerted evolution was reinforced by the large genetic distances between sequences evolving in concert in *T. brucei *and homologs in other species. Additional file 5 shows the ML phylogeny for Tb43 (cysteine peptidase), which is highly conserved within *T. brucei *and other species. Conspecific sequences obviously cluster together, but there is considerable sequence divergence between clades. In fact, the ratio of interspecific to intraspecific divergence was consistently two orders of magnitude greater for these invariant arrays than more variable cases. Tb43 had a ratio of 217.6, while values for Tb26 and Tb75, both moderately variable, were 3.22 and 1.81 respectively. For all of the 'invariant' arrays, the lack of change observed within clades was not matched by the length of branches relating them; these genes had diverged, but not while contained within a single genome.

**Figure 3:**
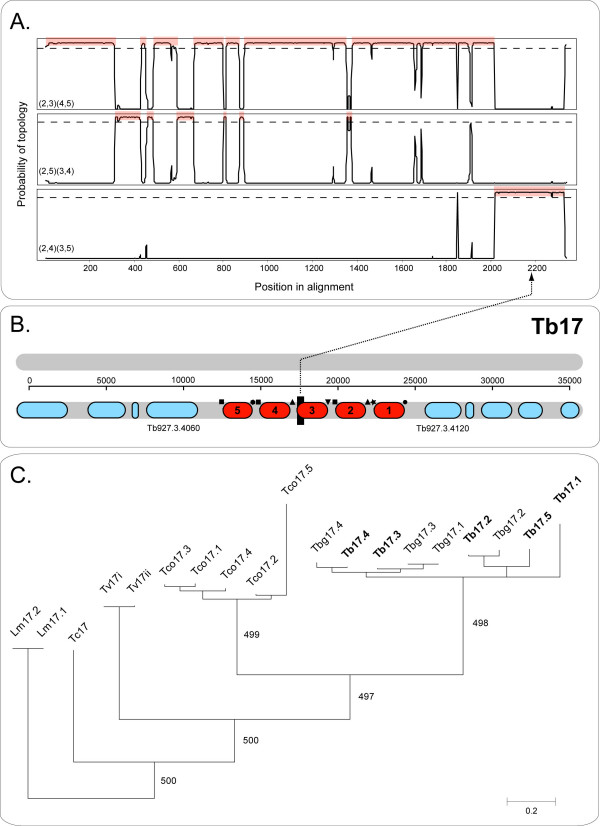
Allelic gene conversion within array Tb17 (hypothetical protein, Tb927.3.4070), identified by phylogenetic criterion. A. TOPALi output showing the results of a sliding window analysis using a HMM method on gene copies 2–5. Phylogenies were estimated for the four sequences in each window along the alignment, producing probabilities for the three possible topologies at each point. Where the probability reaches 1 for any topology (dashed line), the region concerned is shaded. B. Diagram of the chromosomal position of Tb17. The region towards the C-terminus identified as having a significantly different phylogenetic signal, is shown as a black bar and linked to associated evidence in A. C. Phylogenetic tree for homologs of Tb17 from six species. Values are bootstrap proportions out of 500.

Variable arrays also displayed the phylogenetic signal of concerted evolution. Almost all cases for which homoeologous arrays existed in other species produced the pattern of clustering by species. However, this did not extend to *T. brucei *and *T. brucei gambiense*; sequences from these organisms were usually interspersed. The pattern presented by Tb17 in Figure 3 is representative of the general trend; high bootstrap values support species-specific clades and this is confirmed by SH tests, described in Table 1. A topology in which Tb17 sequences group by species is not significantly worse than the most optimal tree (ΔlnL = -10.4, p = 0.448), whereas a topology in which presumed orthologs (based on position) from different species are forced to cluster together is significantly less likely (ΔlnL = -3002.3, p < 0.0001). 16 out of 47 cases failed to reject the concerted evolution scenario and Tb80a confirmed this to the extent that *T. brucei *and *T. b. gambiense *sequences were reciprocally monophyletic also, (the only exception to the previous statement). In 14 out of 47 instances, the cladistic criterion could not be applied because the array was found only in *T. brucei *and *T. b. gambiense*; for these cases it was not possible to infer concerted evolution.

For the remaining tandem arrays, the SH test either rejected the concerted evolution scenario in favour of a pattern of orthology, as for Tb64a; or, as in all other instances, both scenarios were rejected. The optimal tree topology for Tb82 sequences was significantly different to topologies describing concerted evolution and orthology respectively; this occurred because most copies had their closest relative in a different species (i.e., still displayed orthology), while some copies in *T. vivax, T. cruzi *and *L. major *were homogenised or independently derived. Equally, each copy within Tb106 had its closest relative at a different unlinked locus; Tb106 was only represented in *T. brucei, T. b. gambiense *and *T. congolense *and there was no clear indication of the relationships between homoeologous copies. Thus, it is reasonable that this data set should reflect neither concerted evolution nor orthology. A concerted evolution scenario was also rejected by five arrays showing mixed dynamics (Tb24, 64a, 80, 105a, 109c). In these cases the divergent copy retained orthology with homoeologous divergent copies in other organisms (see Figure 1), while the remaining genes in each array were monophyletic. This was sufficient for concert to be rejected, and, conversely, for orthology to be accepted for Tb64a.

In summary, most phylogenies of homologs from various species (alignment 'C', see methods) showed some evidence of concerted evolution, although many arrays could not be tested because they were only present in *T. brucei*. In those that could, monophyly of conspecific gene duplicates was usually significantly more likely than orthology with other species. Even in cases where orthology was retained by certain duplicates, others displayed the signature of concerted evolution.

### Evidence for allelic gene conversion

**Figure 4:**
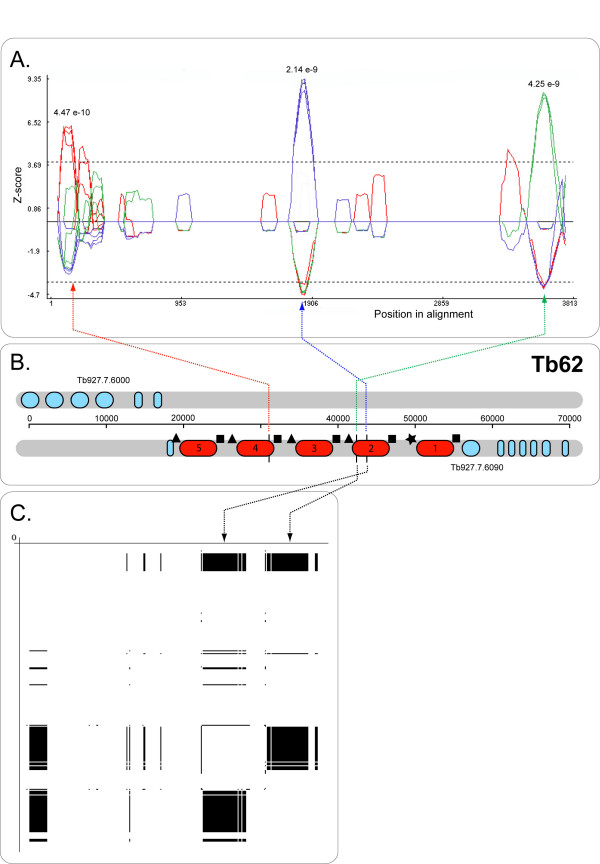
Allelic gene conversion between within array Tb62 (adenylate cyclase, Tb927.7.6080), identified by compatibility criterion. A. SISCAN output, showing the changes in phylogenetic signal along a multiple alignment of gene copies 2, 3 and 4. Genetic distances were calculated to identify the closest relatives from three sequences using a sliding window approach. Coloured lines represent the three phylogenetic resolutions: copies 2 and 3 as closest relatives (red), 2 and 4 (blue) and 3 and 4 (green). The significance of observed similarity is assessed through randomisation of the sequences within a given window and expressed as a Z-score; if the similarity persists over many replicates, outweighing the contribution of any compositional bias, the observation is significant (where the Z-score exceeds 1.96). B. Diagram of the chromosomal position of Tb62. Dashed lines link gene copies to putative breakpoints. C. RETICULATE output. The multiple alignment of all copies is represented by both axes and shaded areas identify where there is incompatibility between the phylogenetic signal of two different regions.

Three different criteria were applied to examine tandem gene duplicates from *T. brucei *only for allelic gene conversion (alignment 'B', see methods). The phylogenetic criterion, applied through the PDM and HMM methods in TOPALi, is illustrated in Figure 3 (Tb17). The distribution criterion, applied through the GENECONV (GCV) method in RDP, is illustrated in Figures 1 and 2 (Tb109c and Tb105a). The compatibility criterion, in the form of RETICULATE, executed in RDP, is illustrated in Figure 4 (Tb62). The consensus of all this information, critically examined by eye, produced a total of 76 GC events, in 21 arrays. These are recorded by sequence in Additional file 6 (Table S1) and by event in Additional file 7 (Table S2).

Like many arrays, Tb17 displayed variation among its duplicate sequences but, despite this, the duplicates clustered together when compared to homoeologs in other species. In addition, Figure 3(A) shows that the pattern of variation was indicative of a gene conversion event at the C-terminus. From here onwards, gene copies will be defined by their position from the 5' end; hence, the third copy of the Tb17 array is written Tb17.3. It follows from Figure 3(C) that Tb17.3 and Tb17.4 were closest relatives overall, an observation supported by the HMM sliding window analysis in Figure 3(A). For the most part, the topology uniting Tb17.3 and 4 attained a probability of 1. However, for a 206 bp section of the 3' UTR, the topology grouping Tb17.3 with Tb17.2 was most likely. Inspection of the multiple alignment suggested conversion of Tb17.3 by Tb17.5, which was most closely related to Tb17.2. This event was supported by GCV (p = 4.62^e-3^) and SISCAN (SSC) (p = 4.88^e-21^) methods.

GCV was successful in identifying three regions in the Tb61 multiple alignment where similarities in the distribution of variation were significant. Additional file 3 (A) shows the output of an SSC analysis, which was executed to confirm the GCV results. When only copies 1, 2 and 8 were compared, three distinct breakpoints were visible from a sliding window analysis that otherwise suggested Tb61.1 and Tb61.2 were always closest relatives. In the left-most anomaly, Tb61.2 and Tb61.8 share significant similarity (p = 2.14^e-9^), which was due to the conversion of Tb61.2 by Tb61.4 between 937 and 1042 bp. The other two regions of significant change refer to instances where Tb61.1 and Tb61.8 were most similar, due to the conversion of copy 1 by 8 between 1393 and 1477 bp (p = 3.41^e-5^), and the conversion of copy 2 by 5 or 7 between 1648 and 1814 bp (p = 4.25^e-12^). Additional files 6 and 7 show that many other events were posited for Tb61, affecting 7/8 duplicates (although PDM/HMM methods only identified events affecting copies 2 and 3).

Different methods for inferring gene conversion were typically concordant; one illustration of this is shown in Figure 4, in which two significant events affecting Tb62.2, 3 and 4 were identified by both SSC and RETICULATE, although a third was not detected by the latter. The left-most peak in Figure 4(A) represents the conversion of Tb62.4 by Tb62.5, causing Tb62.2 and Tb62.3 to be most closely related between 120–450 bp (p = 4.74^e-10^). The other two events refer to, first, Tb62.3 and Tb62.4 clustering together (blue peak, p = 3.38^e-15^), due to conversion of Tb62.2 by Tb62.5, and, second, to Tb62.1 by Tb62.2 being most similar (red peak, p = 3.16^e-09^), also due to conversion of Tb62.2. These two events were identified by the compatibility criterion in panel C, although the precise placement of breakpoints was different.

The majority of putative genetic exchanges were biased, rather than reciprocal. Of 76 observations, only 7 were best explained through the equitable exchange of homologous sequence between two sequences (shown in Additional file 7). All other events involved a given sequence being uncharacteristically similar to unrelated sequences for a given region, and thereby altering its phylogenetic position for the length of that region. These events were interpreted as biased gene conversion.

In many instances, it was possible to delimit the CDS and UTRs within an array, either because EST data was available, or through comparison with the terminal UTRs of the array. Therefore, it was shown that GC frequently affected the UTRs. Tb62 had three putative breakpoints, identified in Figure 4; the event shown at left spanned the start codon, the second event spanned the stop codon, while the peak shown at right corresponded to a region exclusively within the 3' UTR. Similarly, of the 10 GC events identified for Tb93, only three occurred within the coding sequence while another spanned the stop codon.

### Evidence for ectopic gene conversion

The application of GC tests to the 'A' alignments (i.e., tandem gene duplicates from *T. brucei *plus any other gene family members) showed that ectopic gene conversion was very rare among these tandem gene arrays. Only two gene arrays were implicated in EGC by several methods. The first instance concerned Tb105a, where there were significant regions of similarity between the third gene copy and Tb10.70.2860; these loci are separated by 1.17 mb on chromosome 10. Analysis by SSC identified a region between 519–628 bp that showed significant identity (p = 9.88^e-04^), which is indicated by the GCV output in Figure 2(A). These elements of similarity stand out despite Tb105a.3 otherwise being closely related to the first and second gene copies in the array, as shown in Figure 2(C). The second instance of EGC involved Tb32, where the tandem array of 75 KDa invariant surface glycoproteins was preceded upstream by an additional tandem pair of gene duplicates, on the opposite strand. Phylogenetic analysis showed that the tandem pair was most likely to be a segmental duplication of Tb32.3 and Tb32.4. However, while the pair still bore these resemblances, one copy showed regions of similarity to Tb32.2. One of these regions was significant (408–603 bp, p = 1.21^e-11^). Additionally, the tandem pair seemed to have concerted at the N-terminus as a significant GC event was identified here also (136–164 bp, p = 9.61^e-04^).

### Categorisation by evolutionary dynamic and base composition

The tandem arrays in *T. brucei *were categorized according to the evidence presented above, in order to make sense of the various patterns observed and to test the relationship between double strand-break repair mechanisms and base composition (see Additional file 8). Most arrays fell into one of four categories (see methods for explanation): CE-AGC, CE+AGC, AGC and UCO. Exceptions included Tb105a (Figure 2), which was affected by EGC alone, and Tb82, which failed the cladistic criterion (i.e., did not reflect concerted evolution) and retained orthology for all copies. Five arrays fell into the 'mixed dynamics' category, where not all duplicates were monophyletic but a proportion had evolved in concert. Having grouped arrays according to their evolutionary dynamic, the data set was used to evaluate the theory that gene conversion, through double-strand break repair mechanisms, results in G-C enrichment of genomes. The proportion of G-C at third codon positions (G-C3) was calculated for each category and for 100 random, singleton genes from across the genome. This showed that the average G-C3 for singleton genes was 0.416, while the value for UCO arrays was 0.676; this was a highly significant elevation in third position G-C content (df = 10, t = -11.89, p = 1.57^e-07^). A significant elevation was also observed among CE-AGC arrays, where pG-C3 was 0.454 on average (df = 8, t = -2.511, p = 0.018). However, neither the CE+AGC (G-C3 = 0.426) or AGC (G-C3 = 0.435) categories produced significantly different G-C3 to single genes.

## Discussion

Homoeology, the conservation of genomic position, was crucial to establishing the literal equivalence between loci in different organisms, given that these gene sequences were repetitive by nature, and subject to gene conversion that could generate misleading phylogenies. Using this comparative approach, different levels of variation among duplicates were identified among the 47 tandem arrays studied, reflecting various evolutionary dynamics. Where gene duplicates were invariant, UCO was suggested as the mechanism for concerted evolution, but concert was also widespread among variable tandem arrays. The combination of sequence variation and concerted evolution indicated that these arrays were exposed to GC while still diverging. Finally, arrays with copies displaying different patterns in variation were exceptions that proved the rule: copies within homogeneous repeat structures conformed to a pattern indicative of concerted evolution, while others physically segregated from the repetitive region retained orthology with loci in other species.

### Patterns of sequence variation indicative of concerted evolution

For most arrays that could be tested the cladistic criterion firmly rejected orthology between homoeologous gene copies in different organisms. For tandem arrays present only in *T. brucei *it was not possible to find evidence for concerted evolution, given that lack of variation itself is not evidence. If one accepts that where there is conserved gene order around a homologous array in multiple genomes, this proves that the loci are orthologous (i.e., directly inherited from an ancestor with an equivalent character), the failure of these orthologs to appear monophyletic in a phylogeny is evidence for the alteration of conspecific sequences, i.e., concerted evolution within individual genomes. If one does not accept this as the cause of the unexpected homogeneity, convergence due to positive selection or, more plausibly, the removal of substitutions due to strong structural conservation (i.e., purifying selection) are possible alternatives.

It has been suggested that a mixture of recent gene duplication and purifying selection can explain why tandem gene duplicates appear similar [5]. In this study, purifying selection is not a sufficient explanation since the gene copies have evolved; including those 'invariant' arrays showing large genetic distances between species. Whatever small amount of variation exists should still reflect the orthologous relationships indicated by shared genomic position. Therefore, purifying selection would only be adequate if posited after the isolation of a genome, coinciding with the origin of an array by gene duplication *de novo*. Rapid gene duplication would then result in homogenous gene copies, clustering by species in a phylogeny. Yet, many arrays in this study were clearly not exposed to purifying selection and were evolving neutrally; even invariant arrays displayed large interspecific genetic distances and so had diverged within each lineage. And it is implausible that an array present in five different species was inherited by each as a single gene and was independently expanded five times. It is more parsimonious that the array present in all species was an array in their ancestor.

Among the 'invariant' tandem arrays, concerted evolution was the result of unequal crossing-over between homologous chromosomes at meiosis. The absence of any variation within the non-coding parts of the arrays, near-zero *D*_*n *_and *D*_*s *_values in the CDSs and, conversely, substantial divergence from homologs in other species, suggest that variation has been purged from these sequences irrespective of codon position, rather than constrained by purifying selection. In other words, while UCO results in dispersal of polymorphisms among gene duplicates and thus 'invariant' arrays within individual genomes, divergence between homologs in different genomes continues. In agreement with previous observations, the G-C3 content is significantly elevated among these arrays, supporting the theory that double-strand break repair mechanisms cause G-C enrichment [45,46]. In *T. brucei*, homogeneity has previously been observed among the highly-conserved heat-shock proteins [29,47]. These and other arrays in *Trypanosoma *spp., such as histones [48,49] and cysteine protease [50,51], emulate the homogeneity among important structural or 'housekeeping' genes in other organisms [9].

The idea that purifying selection could explain concert among arrays with greater levels of variation is even more unlikely, given that non-coding regions were once again often more conserved, and many genes were evolving neutrally. The coincidence of variation and concerted evolution demands a different mechanism: gene conversion. These arrays suggest that repeated gene conversion can cause tandem duplicates to evolve in concert, in the presence of neutral divergence (or even positive selection). Although no GC was observed in 7 arrays where concerted evolution was inferred under the cladistic criterion, the incidence of GC suggests that both homogenising and diversifying processes operate simultaneously within these genomic structures. It is not clear which would occur more frequently, or where the balance of forces would lie; however, increased divergence would inevitably reduce the probability of conversion between two sequences and less conservative regions of a gene may rapidly escape conversion after duplication, allowing unfettered divergence.

In summary, the action of concerted evolution in tandem arrays is betrayed by the loss of orthology between homoeologous genes in different organisms, yet this does not seem to prevent divergence between and within genomes. These results show that paralogous sequences can become more similar over time, even in the presence of divergence, and that such concerted evolution is only one conservative process affecting duplicate sequences, which may be simultaneously affected by other conservative or innovative forces.

### Allelic gene conversion as a mechanism of sequence evolution

21 out of 47 tandem arrays contained chimaeric gene copies, which generated putative cases of partial gene conversion. In the majority of cases this gene conversion was biased and presumably occurred with the effect of propagating one sequence motif at the expense of another. On a few occasions, recombination between two sequences was the best explanation for incompatible motifs in an alignment. The extent of AGC may be difficult to determine since it cannot be known how many exchanges have been 'overwritten' by subsequent events. And as no distinction was made between homologous chromosomes in the *T. brucei *genome sequence, it is not known what proportion of events occurred between alleles during meiosis. The authenticity of these putative GC events was checked by collecting voucher sequences from those read libraries that were available (chromosomes 9, 10 and 11). Of all GC events originating on these chromosomes, only four could not be substantiated by single read sequences for three gene copies containing incompatible motifs (see Additional file 7). For these cases, the contradiction between one part of the molecule and another could be due to the assembly of individual reads from different copies. However, single reads were retrieved for most putative events that covered the breakpoint and represented both incompatible motifs, eliminating the possibility of such mis-assembly.

AGC is a plausible mechanism for homogenisation of many of these tandem arrays. However, in certain circumstances, where sequences diverge rapidly, AGC may have a further diversifying effect by helping to disperse sequence motifs among duplicates. Tb93 and Tb17, both coding for surface-expressed proteins, may be examples of where the rate of AGC is outstripped by sequence divergence, preventing a net homogenising effect of GC (although, by sharing around sequence motifs, array copies would still cluster by species in a phylogeny). Contingency gene systems are a dramatic illustration of the power of GC to generate allelic diversity through the creation of chimaeric sequences. Contingency genes have evolved independently in various pathogens and parasites to provide a rapid solution to immunity raised against an existing epitope. Typically they comprise a large number of whole and partial surface antigens, only a fraction of which are expressed. A novel gene is generated by transposing an inactive motif into an expression locus, replacing the existing sequence, for example, in the generation of novel *pilin *in *Neisseria *[52], translocation of *VSG *pseudogenes into expression sites in *T. brucei *[53,54] and similarly in *Babesia bovis *[55] and the cestode *Echinococcus *[56]. These systems employ EGC in the generation of chimaeras; this, and the concerted evolution of conserved gene families, such as β-tubulin in *Leishmania *[57], suggested *a priori *that EGC played a prominent role in the evolution of gene duplicate sequences across the genome.

However, in contrast to AGC between tandem duplicates physically associated within an array, exchange between gene family members in *trans*, or separated from the array, was seldom observed. It is reported that conversion frequency is lower between distant and dissimilar gene copies [37,38,58,59], which suggests that gene conversion is unlikely to affect the sequence homogeneity of gene families in general. Only two arrays, Tb32 and Tb105a, produced a substantiated incidence of EGC, despite initially promising patterns in Tb12 and Tb26. The latter two demonstrated the difficulty in confirming gene conversion events; the presence of recently duplicated gene copies near to the tandem array gave the impression of gene conversion with array copies. However, any similarity between genes in and out of the array was interpreted as common inheritance from a duplication event, and any incompatibilities were introduced subsequently by allelic gene conversion between newly duplicated copies rather than through ectopic exchange with the array.

### Functional differentiation and the spatial context of concerted evolution

UCO and GC are recognised mechanisms of concerted evolution [9]. Both processes occur due to the aberrant alignment of repetitive sequences in *cis *or *trans*. Since these processes involve the interaction of repetitive sequences, the frequencies of UCO and GC decline with physical distance and sequence dissimilarity [60,61], which is corroborated by the paucity of EGC here. The limitations on concerted evolution occur because it is an explicitly spatial phenomenon [62], for instance, it is largely limited to tandem arrays in *T. brucei*, and does not extend to gene families in general. In this study, the structural limitations on concerted evolution are very well demonstrated by arrays with mixed dynamics. While gene duplicates in arrays such as Tb109c and Tb24 show physical proximity and sequence homology typical of other arrays, the repeat structure of the array is disrupted through extensive divergence in flanking and, sometimes, coding sequences. In Additional file 4, Tb24.4 and 5 have evolved unique UTRs and are physically segregated from Tb24.1-3 by the insertion of two unrelated genes; perhaps as a consequence, they retained orthology with homoeologs in other species. Similarly, Tb109c.4 (Figure 1) is physically separated from the array by an unknown CDS and is shown to retain orthology with homoeologs in *T. cruzi, T. congolense *and *L. major*. And likewise, Tb105a.4 (Figure 2) is segregated from the other, concerted duplicates by its UTRs, which show no affinity with others in the array, and is more closely related to other genes around the genome. These cases link the disruption of the repeat structure, by rearrangements to IGS or CDS boundaries, with divergent gene copies that retain orthology across species, rather than converging with conspecific paralogs.

Previous studies have shown that tandem arrays have the capacity to harbour important functional variation. The phosphoglycerate kinase (PGK) array in *T. brucei *comprises three distinct sequence types, where the second copy is a chimaera of the first and third, created through gene conversion [63,28]; this variation is responsible for the expression of specific glycosomal and cytoplasmic isoforms [64,65]. In a similar instance, the hexose transporter array in *T. brucei *reflects the spatial and functional partition of two different isoforms, each with several identical duplicates, which may again have been affected by gene conversion between the isoforms [30]. In *T. cruzi*, duplicates within the *pyr *gene array controlling pyrimidine biosynthesis are also functionally distinct and expressed in particular life-cycle stages [66]. Sequence variation in the 3' UTRs of β-tubulin in trypanosomatids has been directed related to differential regulation of gene expression in different life stages [67,68]. Hence, given the abundance of sequence variation evident here, functional differentiation of tandem duplicates may be commonplace, suggesting a subtle functionality within tandem arrays not yet appreciated in *T. brucei*. The presence of both gene duplicates evolving in concert and others retaining orthology in mixed dynamic arrays demonstrates how gene conversion is a spatial phenomenon. Rearrangements of the primary or secondary structures of divergent gene copies make them permanently distinct and reduce the probability of recombination with a nearby duplicate. This offers a model for how functional differentiation can be reconciled with the ubiquity of concerted evolution observed here.

## Conclusion

Contrary to many previous descriptions from trypanosomatids, tandem gene arrays in *T. brucei *contain substantial genetic variation among gene duplicates. Despite this, concerted evolution is pervasive, and evidence from various loci indicates that both unequal-crossing over and allelic gene conversion contribute to homogenisation. Therefore, gene duplicates can both evolve in concert, relative to homoeologs in other species, and diverge within an array. This indicates that multiple evolutionary forces are apparent, with sequence variation determined by a balance of conservative and innovative pressures. Structurally heterogeneous tandem arrays demonstrated that the influence of concerted evolution is mediated by the spatial environment of gene duplicates; it can be prevented by disruption of the repeat structure in the array. Functional differentiation of duplicates can and does occur through such rearrangements in intergenic sequences. Together, the dynamics of sequence variation among tandem duplicates show how various forces affecting gene sequences must be integrated with typical 'birth-and-death' ideas of gene family evolution, to produce an adequate model. Abundant variation among gene duplicates indicates that tandem duplication has greater consequence than simply increasing gene dosage; the systematic account given here provides a basis for uncovering the hidden functionality within tandem gene arrays in trypanosomatids.

## Methods

The primary resource for all analyses was the completed genome sequence for *Trypanosoma brucei *[6], which was available from the Wellcome Trust Sanger Institute's interface 'GeneDB' [69]. When evaluating concerted evolution, the completed sequences of *Trypanosoma cruzi *[70] and *Leishmania major *[44], and the draft genome sequences for other trypanosomatids (*T. brucei gambiense, T. congolense, T. vivax*), were also utilised through the same interface. The *T. brucei *genome consists of 11 chromosomes ranging between 1 and 5 mb. Each chromosome was scanned by eye for duplicate genes positioned in tandem; where the similarity was not obvious from annotation suspected duplicates were tested by reciprocal BLAST and sequence alignment. A catalogue of all tandem gene pairs and gene arrays is available as supplementary material (see Additional file 9).

Since this study concerned the variation among gene copies and the approach taken was inherently phylogenetic, only those tandem arrays with >3 copies in the annotated genome sequence were selected for analysis; these included tandem segmental duplications where the repeat units comprised multiple dissimilar, contiguous genes. The component coding sequences (typically, trypanosomatid genes do not contain introns, although they do occasionally occur [71]) for each duplicated locus were then extracted. The protocol for analysis of sequence variation is described in Additional file 8. Essentially, after preparation of multiple alignments, the variation among sequences was quantified and characterised using phylogenetic estimation. The *cladistic, phylogenetic, distribution *and *compatibility *criteria described previously were applied to detect concerted evolution, AGC and EGC in turn. Each array was then categorised according to the results of these analyses to examine the relationship between evolutionary dynamic and base composition.

### Data preparation

Duplicate sequences were aligned using the ClustalW tool [72] for each tandem array, within the BioEdit platform [73]. Sequences were translated prior to alignment to preserve codon structure and then returned to nucleotide form for analysis. Each alignment was checked by eye. Three types of alignment were created:

• A: duplicate sequences from the array were combined with all other gene family members. Untranscribed regions (UTRs) were not included because they usually could not be aligned. These alignments were intended to test for monophyly of array copies and to test for ectopic exchange between unlinked loci in the same gene family. These alignments were not possible in 15/47 cases because no further loci existed beyond the tandem array.

• B: the coding sequence and UTRs of each copy from the array were aligned. UTRs were defined using either expressed sequence tag (EST) data, where this existed, or on the basis of identity between internal and external intergenic sequences (IGS). The first and last copies of the array usually had curtailed (sometimes entirely different) 5' and 3' non-coding regions respectively. The length over which these regions displayed identity with the internal IGS was assumed to reflect the UTR. These alignments were created to estimate variation and phylogeny among array copies and to evaluate the evidence for allelic gene conversion.

• C: the coding sequence for each array copy in *T. brucei *was combined with homologs from up to five other species, where available. UTRs were not included because these often do not align over large phylogenetic distances. Homologs were found by searching each genome sequence with the gene sequence of surrounding genes in *T. brucei*. For a homolog to be accepted, it had to display 'homoeology' with *T. brucei*, i.e., conservation of surrounding gene order.

### Analysis of concerted evolution

Variation among duplicate sequences within arrays was quantified with the numbers of synonymous (*D*_*s*_) and non-synonymous (*D*_*n*_) substitutions per site, using DNAsp [74]; these measures were favoured because they reflect purifying selection, an alternative to UCO in explaining low divergence. Concerted evolution should result in a clade of tandem duplicates, excluding homologs in other species. Hence, the cladistic criterion was applied by estimating a phylogenetic network for Alignment C. A network has advantages as an initial analysis because ambiguity in a phylogenetic hypothesis remains explicit, but it is also realistic where sequences have mixed histories (as many genes may have among these data sets). The networks were estimated using the neighbour-net method, a clustering algorithm executed in Splitstree v4.0 [75]. To correct for potential base composition bias, a logdet genetic distance matrix was used after removing parsimony-uninformative sites.

Where all array copies were monophyletic, this was confirmed by estimating maximum likelihood phylogenetic trees using PHYML [76], with 500 non-parametric bootstraps. The trees were unrooted, a GTR + I + Γ model was applied and parameters were estimated from the data [77]. Statistical evaluation of the concerted evolution hypothesis was obtained using a Shimodaria-Hasegawa test (SH, [78]), applied to alignment C; the test was executed using DNAml in the PHYLIP 3.65 package [79]. The SH test compared the most optimal topology without constraints with two tree topologies in which duplicate sequences were monophyletic by species (i.e., concerted) and by position in the array (i.e., retained orthology) respectively. The 'concerted' and 'orthologous' scenarios could be rejected where there was a significant departure in the likelihood between optimal and constrained topologies. Failure to reject the null hypothesis that the constrained topology was sub-optimal showed that any apparent differences were explicable due to sampling error.

### Analysis of allelic gene conversion

AGC is defined as recombination between gene duplicates within an array, either between copies on the same chromosome or between homologous chromosomes. *Phylogenetic, compatibility *and *distribution *criteria were applied in detecting AGC events. All putative GC events were checked by eye and many were rejected when related back to the multiple alignment.

The phylogenetic criterion detected GC by identifying a significant difference in tree topology between neighbouring 'windows' in a sliding window analysis. This was conducted using the TOPALi platform [80], with the probabilistic divergence measure (PDM, [81]) and hidden Markov model (HMM, [82]) methods. The PDM method scans the entire alignment, estimating phylogenies for fixed sequence windows with a Markov Chain Monte Carlo process. A significant change in the posterior probabilities of two neighbouring windows is interpreted as a breakpoint. A step size of 1 and window size of 10 were applied, with otherwise default settings. Possible breakpoints were further analysed using the HMM method, which detects recombination breakpoints among four sequences by modelling the probabilities of transitions between the three tree topologies. This determines which topology, at any point along the alignment, is most probable. Both PDM and HMM models are designed to compare tree topologies only and minimise the bias introduced by rate heterogeneity among and within sequences [80].

The compatibility criterion detected breakpoints by identifying neighbouring sites in the multiple alignment that gave incompatible phylogenetic signals. These sites were plotted on to a 2-dimensional space using the program RETICULATE [35], within the RDP v2.0 platform [83]. Only binary sites were used and 100 randomised matrices were estimated. These breakpoints were again used to inform subsequent methods of GC detection.

The distribution criterion detected regions of significant similarity between sequence pairs by comparing the distribution of silent polymorphisms along the alignment, i.e., those changes unaffected by purifying selection. Significant clustering of identical polymorphisms was identified by GENECONV (GCV, [84]), executed within RDP v2.0. The analysis compared sequence triplets, using default settings. P-values for GC events were calculated by permutation of the data set, with 1000 randomisations. GENECONV produced a large number of candidate gene conversions, but many of these were created by rate differences within and among sequences. Each candidate event was checked to remove the obvious mistakes caused by regions of high conservation or derivation. Furthermore, each event was checked using a second method in the RDP package, SISCAN (SSC, [85]). This method conducts a sliding window analysis with a phylogenetic criterion, calculating a p-value through sequence permutation. Default settings were applied.

Putative AGC events required validation. Tandem gene arrays are among the most challenging parts of genome sequences to assemble, for much the same reason that non-coding repetitive regions are difficult to resolve; assembling sequence reads into contigs relies on unique motifs to define position. It is especially difficult to determine the precise number and order of copies within an array, where the copies differ by less than 5%. Consequently, in tandem arrays with low variation, gene copies may be mis-assembled and appear chimaeric because parts of different copies have been conjoined. To consider this, the original sequence reads were collected for every three sequences involved in a putative GC event on chromosomes 9, 10 and 11, to act as vouchers. If sequence X and Y were closest relatives, except for one region where X was more akin to Z, the appropriate regions of all three sequences had to be represented in sequence reads (each covering 0.5–1 Kb) to confirm that a phylogenetic conflict truly existed.

### Analysis of ectopic gene conversion

Where alignment A was available, tests for GC were repeated as described above for each array. These comparisons were designed to identify significant similarity between gene duplicates at different loci that may be caused by EGC. Putative EGC events were validated by collecting voucher sequences as before, although the potential for mis-assembly is reduced for single-copy genes.

### Relationship between gene conversion and base composition

*T. brucei *tandem arrays were classified according to the results of the preceding tests. The categories shown in Additional file 8 classify arrays by the presence or absence of AGC in conjunction with concerted evolution (CE+AGC and CE-AGC arrays); the presence or absence of EGC in conjunction with concerted evolution (CE+EGC and CE-EGC arrays), those with some duplicates retaining orthology, and others concerted ('mixed dynamics'); those unique to *T. brucei *and showing AGC; those that have retained orthology; and those with identical copies within *T. brucei *but divergence between species (i.e., UCO or 'invariant' arrays). To test the hypothesis that double strand-break repair mechanisms are biased towards guanine-cytosine (G-C) and result in G-C enrichment of regions undergoing recombination or biased gene conversion [45,46], the average G-C content at the third codon position (G-C3) of each class of tandem array was calculated using the base composition function in GCUA [86]. This was then compared to the average G-C3 value for 100 singleton genes chosen at random from all chromosomes.

## Supplementary Material

Additional File 1 **Figure S1**. Models of unequal crossing-over (UCO) and gene conversion (GC). A. UCO. A tandem array of gene duplicates is shown on homologous chromosomes; colours denote slight differences in sequence. Due to the repetitive structure of the array, mis-alignment between alleles during meiosis or between chromatids during mitosis is frequent. This results in unequal crossovers between homologous chromosomes that change the length of the array and gradually homogenise it, as particular sequence types are lost by chance. B. GC. Non-reciprocal genetic exchange between two gene duplicates can occur in a variety of ways, five of which are shown here. Four gene duplicates are present in a tandem array on chromosome 1; these have slight differences in sequence reflected by colour. Another single locus is shown on chromosome 2. For both chromosomes 1 and 2, homologous pairs are shown and depicted after DNA synthesis but before segregation, i.e., each has two sister chromatids. In each of the following scenarios, gene conversion might be complete or partial: i. intrachromatid, ii. interchromatid, iii. allelic, iv. allelic (partial), v. ectopic. In this study, types i.-iv. are all been referred to as allelic gene conversion (AGC) as they are only distinguishable from type v., ectopic gene conversion (EGC). Click here for file

Additional File 2 **Figure S2**. Phylogenetic relationships of gene duplicates within Tb55 (retrotransposon hot-spot protein, Tb927.7.2030). A. Phylogenetic network showing all possible genetic distances between all gene duplicates. Numbers refer to bootstrap proportions out of 100. B. Diagram of the chromosomal position of Tb55. Grey bars represent chromosomal strands and are measured in base pairs. Named CDS are coloured green and may be given GeneDB identifiers. Hypothetical CDS are coloured blue. Array copies are coloured red and numbered as they appear in the text. The affinity of UTRs is denoted by black symbols adjacent to each gene duplicate. Identical symbols denote alignable non-coding sequences. C. Phylogenetic network of gene duplicate 5' UTR sequences. D. Phylogenetic network of gene duplicates 3' UTR sequences. Click here for file

Additional File 3 **Figure S3**. Allelic gene conversion within array Tb61 (hypothetical protein, Tb927.7.5930), identified by phylogenetic criterion. A. SISCAN output, showing the changes in phylogenetic signal along a multiple alignment of gene copies 1, 2 and 8. The likelihood of each phylogenetic grouping is expressed as Z-score and shown as coloured lines: copies 1 and 2 as closest relatives (green); 2 and 8 (red); 1 and 8 (blue). Three putative gene conversion events are marked with associated p-values and arrows linked to position in the array. B. Diagram of the chromosomal position of Tb61. Dashed arrows link evidence for gene conversion with the associated chromosomal positions. C. Phylogenetic tree for gene copies from *T. brucei *and *T. b. gambiense*. Click here for file

Additional File 4 **Figure S4**. Mixed patterns of variation within array Tb24 (serine/threonine-protein phosphatase, Tb927.4.3640). A. Diagram of the chromosomal position of Tb24. B. Phylogenetic tree for Tb24 homologs from four species; orthology is retained by the divergent fourth and fifth duplicates, producing a distinct clade (shaded). Click here for file

Additional File 5 **Figure S5**. Concerted evolution among array Tb43 (cysteine peptidase, Tb927.6.1060), identified by cladistic criterion. A. Diagram of the chromosomal position of Tb43. B. ML phylogenetic tree showing all gene copies from *T. brucei *and homologs from four other species. Values are bootstrap proportions out of 100. Click here for file

Additional File 6 **Table S1**. Gene conversion events catalogued by tandem gene array. Click here for file

Additional File 7 **Table S2**. Gene conversion events catalogued by sequence individual gene duplicate sequence Click here for file

Additional File 8 **Figure S6**. Flowchart showing the various stages of the study protocol. The four criteria used in tests for concerted evolution and gene conversion are capitalised and the programs employed are bracketed. Tandem arrays were classified according to the results of these tests and an overall 'interpretation' was made regarding their dynamics (see methods); arrays are listed under each category with their identifier tag, as specified in Table 1. Click here for file

Additional File 9 **Table S3**. Identity and position of tandem gene pairs and arrays in *T. brucei*. Click here for file
