# Supplementary figures and images for: Tandem gene arrays in Trypanosoma brucei: Comparative phylogenomic analysis of duplicate sequence variation
Source: BMC Evol Biol. 2007 Apr 4;7:54. doi: 10.1186/1471-2148-7-54 (PMC1855330; doi:10.1186/1471-2148-7-54)

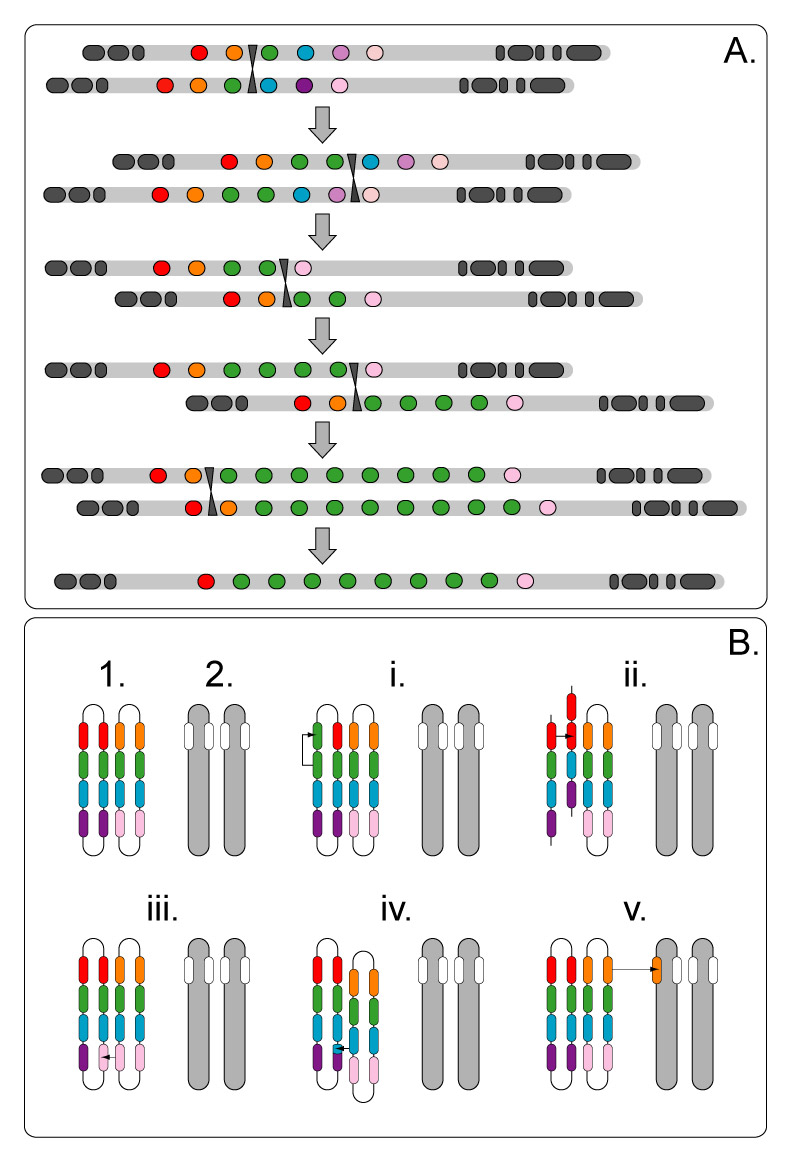

Supplement: Additional File 1 — Figure S1. Models of unequal crossing-over (UCO) and gene conversion (GC). A. UCO. A tandem array of gene duplicates is shown on homologous chromosomes; colours denote slight differences in sequence. Due to the repetitive structure of the array, mis-alignment between alleles during meiosis or between chromatids during mitosis is frequent. This results in unequal crossovers between homologous chromosomes that change the length of the array and gradually homogenise it, as particular sequence types are lost by chance. B. GC. Non-reciprocal genetic exchange between two gene duplicates can occur in a variety of ways, five of which are shown here. Four gene duplicates are present in a tandem array on chromosome 1; these have slight differences in sequence reflected by colour. Another single locus is shown on chromosome 2. For both chromosomes 1 and 2, homologous pairs are shown and depicted after DNA synthesis but before segregation, i.e., each has two sister chromatids. In each of the following scenarios, gene conversion might be complete or partial: i. intrachromatid, ii. interchromatid, iii. allelic, iv. allelic (partial), v. ectopic. In this study, types i.-iv. are all been referred to as allelic gene conversion (AGC) as they are only distinguishable from type v., ectopic gene conversion (EGC). [file 1471-2148-7-54-S1.jpeg]

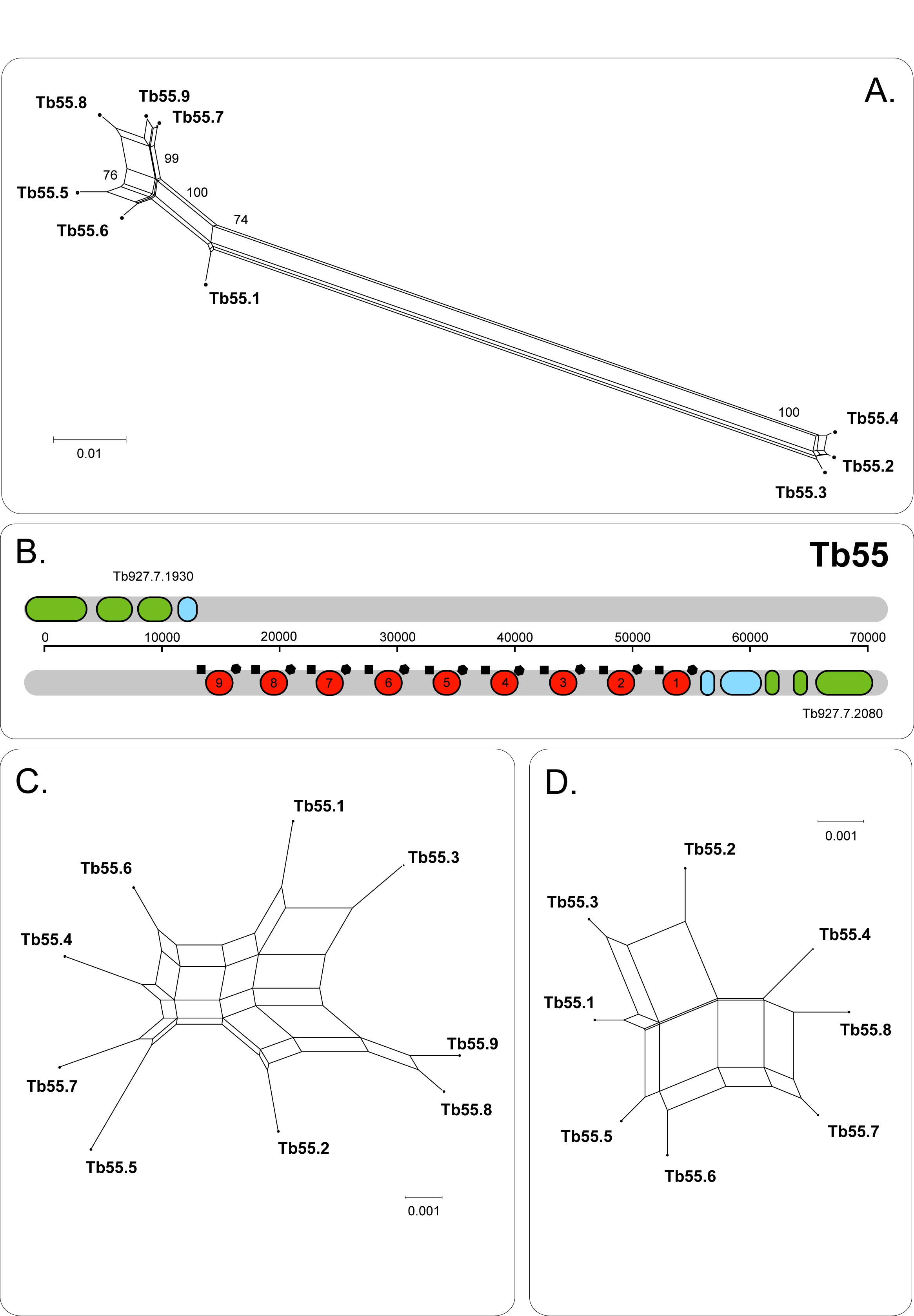

Supplement: Additional File 2 — Figure S2. Phylogenetic relationships of gene duplicates within Tb55 (retrotransposon hot-spot protein, Tb927.7.2030). A. Phylogenetic network showing all possible genetic distances between all gene duplicates. Numbers refer to bootstrap proportions out of 100. B. Diagram of the chromosomal position of Tb55. Grey bars represent chromosomal strands and are measured in base pairs. Named CDS are coloured green and may be given GeneDB identifiers. Hypothetical CDS are coloured blue. Array copies are coloured red and numbered as they appear in the text. The affinity of UTRs is denoted by black symbols adjacent to each gene duplicate. Identical symbols denote alignable non-coding sequences. C. Phylogenetic network of gene duplicate 5' UTR sequences. D. Phylogenetic network of gene duplicates 3' UTR sequences. [file 1471-2148-7-54-S2.jpeg]

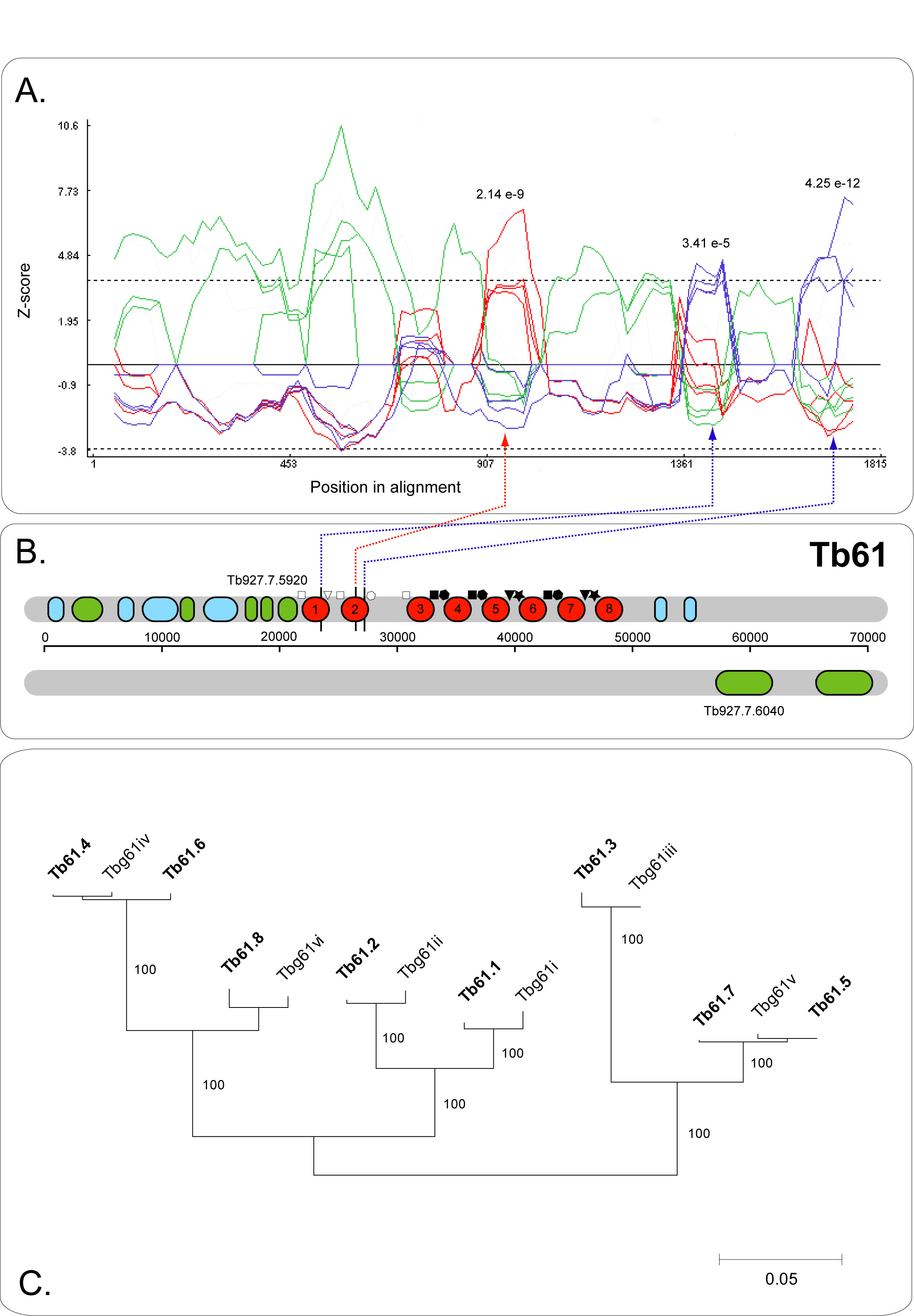

Supplement: Additional File 3 — Figure S3. Allelic gene conversion within array Tb61 (hypothetical protein, Tb927.7.5930), identified by phylogenetic criterion. A. SISCAN output, showing the changes in phylogenetic signal along a multiple alignment of gene copies 1, 2 and 8. The likelihood of each phylogenetic grouping is expressed as Z-score and shown as coloured lines: copies 1 and 2 as closest relatives (green); 2 and 8 (red); 1 and 8 (blue). Three putative gene conversion events are marked with associated p-values and arrows linked to position in the array. B. Diagram of the chromosomal position of Tb61. Dashed arrows link evidence for gene conversion with the associated chromosomal positions. C. Phylogenetic tree for gene copies from T. brucei and T. b. gambiense. [file 1471-2148-7-54-S3.jpeg]

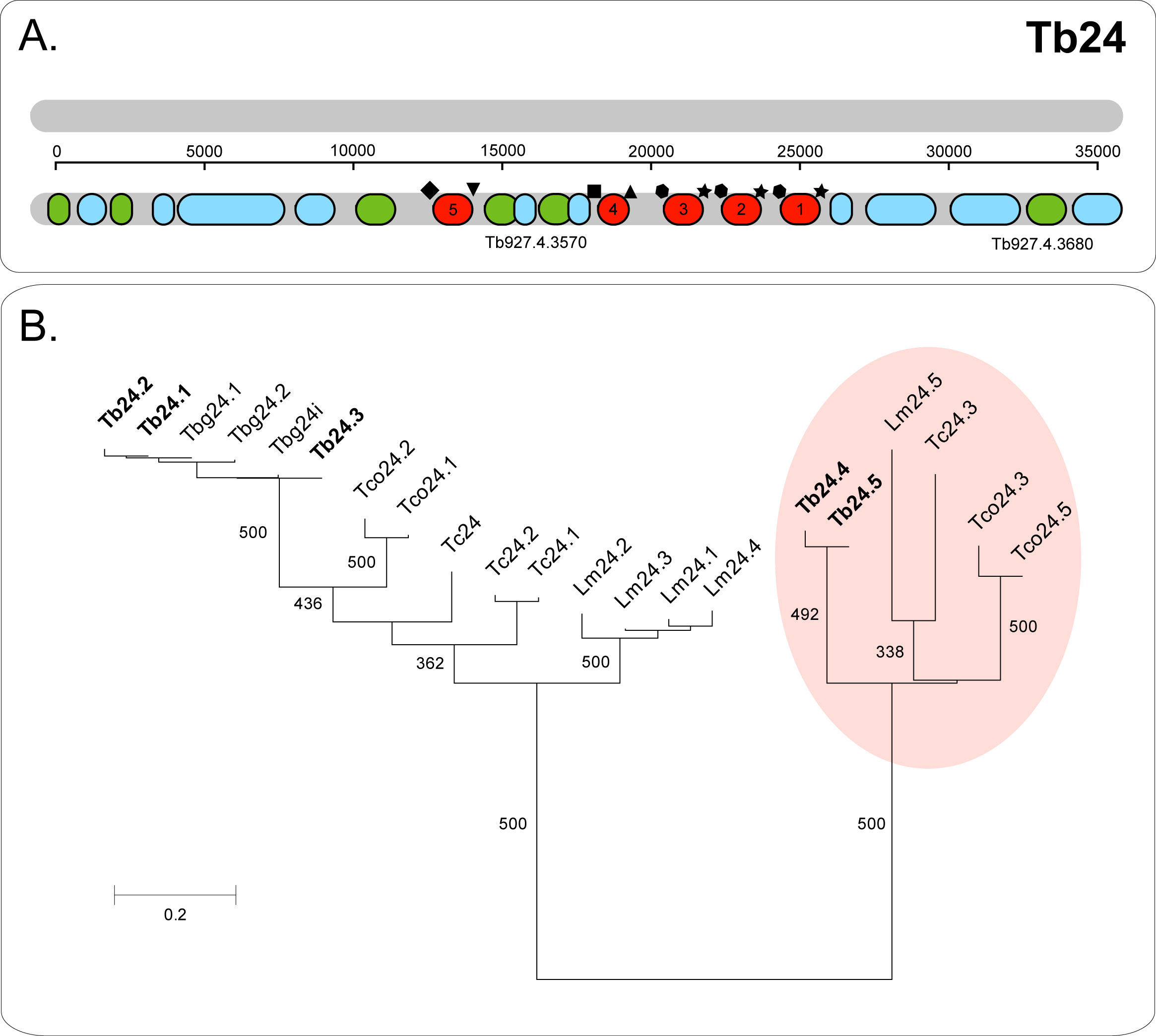

Supplement: Additional File 4 — Figure S4. Mixed patterns of variation within array Tb24 (serine/threonine-protein phosphatase, Tb927.4.3640). A. Diagram of the chromosomal position of Tb24. B. Phylogenetic tree for Tb24 homologs from four species; orthology is retained by the divergent fourth and fifth duplicates, producing a distinct clade (shaded). [file 1471-2148-7-54-S4.jpeg]

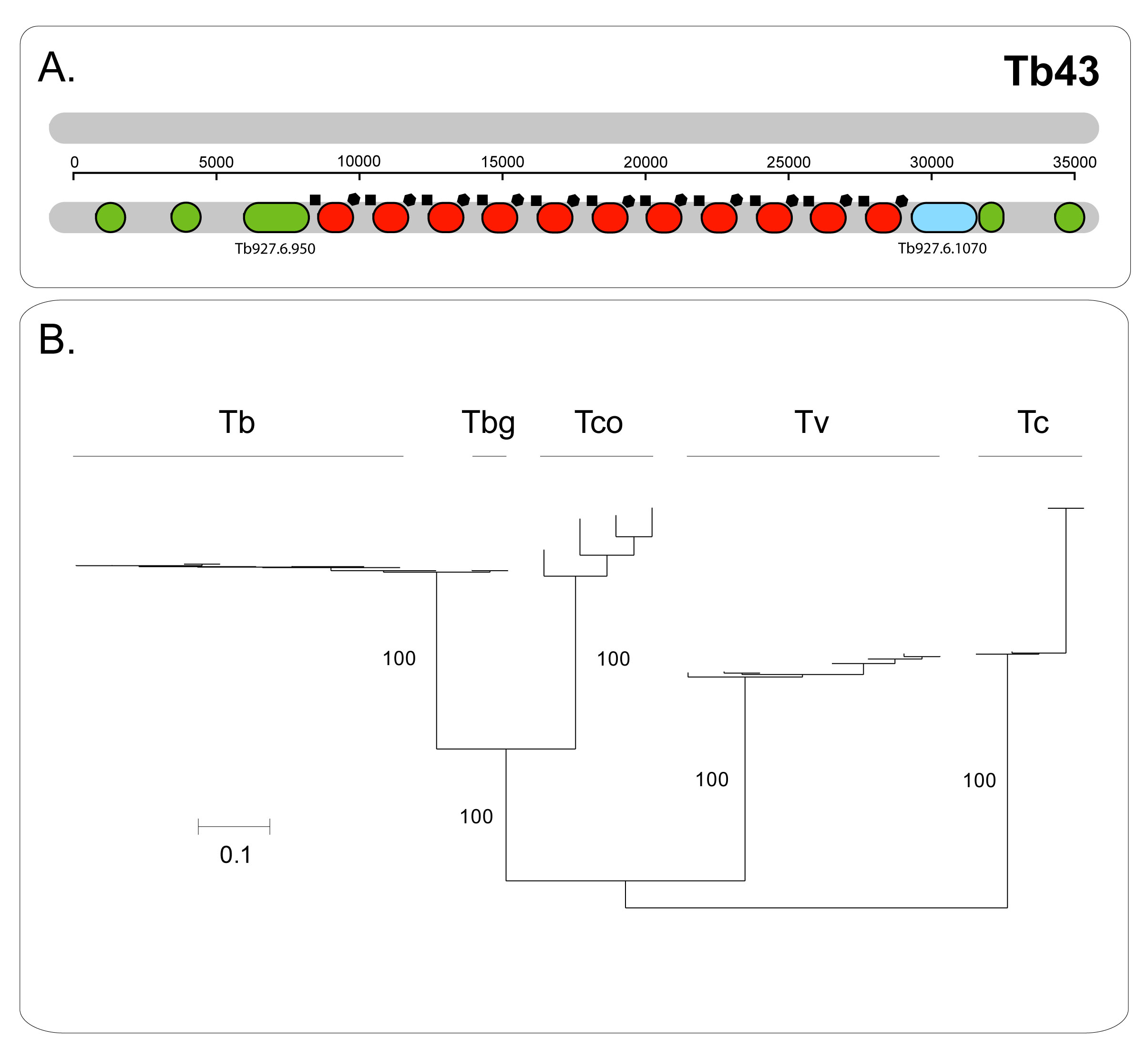

Supplement: Additional File 5 — Figure S5. Concerted evolution among array Tb43 (cysteine peptidase, Tb927.6.1060), identified by cladistic criterion. A. Diagram of the chromosomal position of Tb43. B. ML phylogenetic tree showing all gene copies from T. brucei and homologs from four other species. Values are bootstrap proportions out of 100. [file 1471-2148-7-54-S5.jpeg]

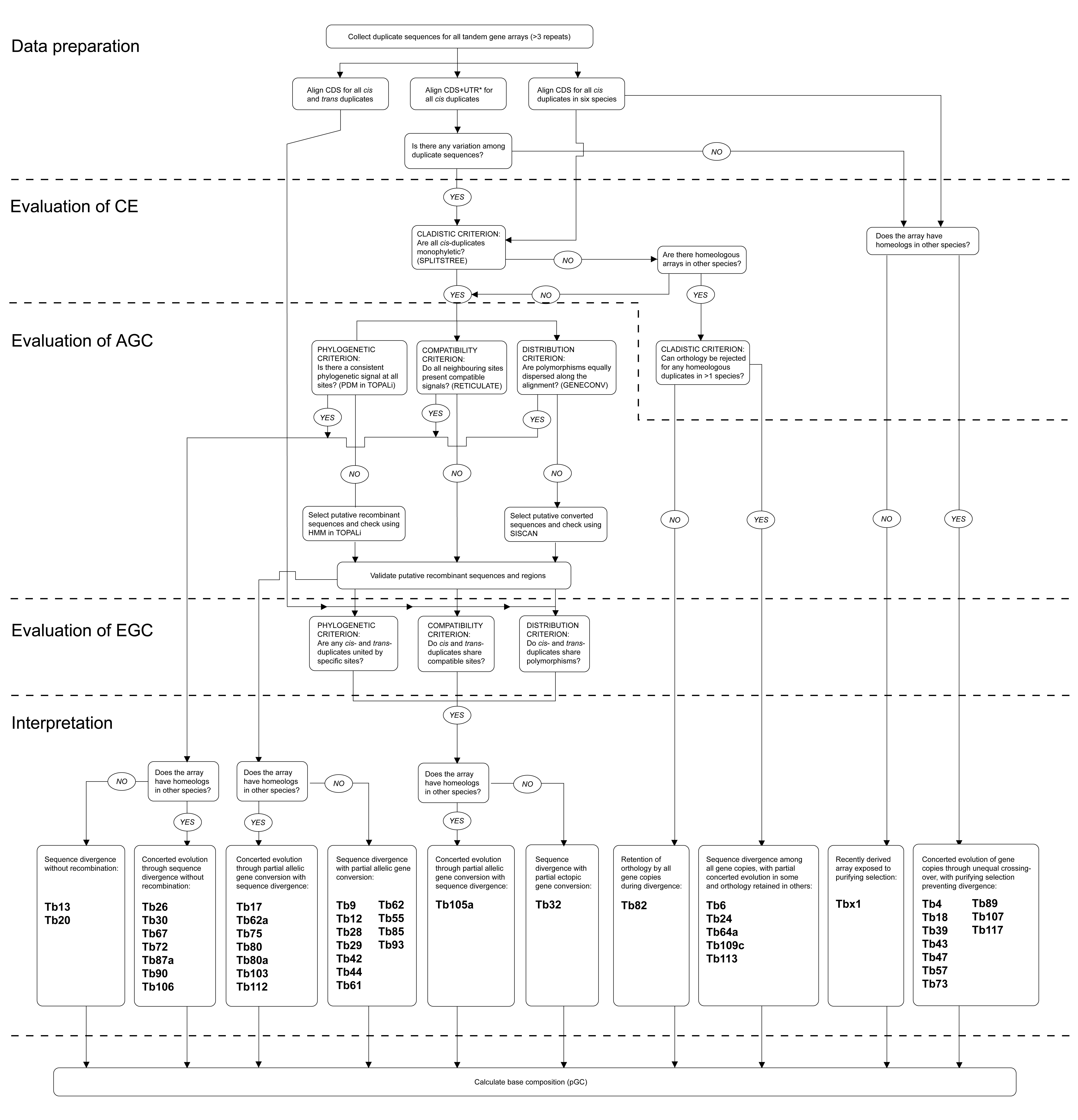

Supplement: Additional File 8 — Figure S6. Flowchart showing the various stages of the study protocol. The four criteria used in tests for concerted evolution and gene conversion are capitalised and the programs employed are bracketed. Tandem arrays were classified according to the results of these tests and an overall 'interpretation' was made regarding their dynamics (see methods); arrays are listed under each category with their identifier tag, as specified in Table 1. [file 1471-2148-7-54-S8.jpeg]
