# Supplementary material for: Tandem gene arrays in Trypanosoma brucei: Comparative phylogenomic analysis of duplicate sequence variation
Source: BMC Evol Biol. 2007 Apr 4;7:54. doi: 10.1186/1471-2148-7-54 (PMC1855330; doi:10.1186/1471-2148-7-54)
Supplement: Additional File 6 — Table S1. Gene conversion events catalogued by tandem gene array. [file 1471-2148-7-54-S6.doc]

**Table S1. Gene conversion events and G-C3 content by array.**

| ID | Description | Copy | pGC3 | Parent/Donor sequences: | | | | | | | |  |  |  |  |  |  |  |  |  |  |  |  |  |  |  |  |  |  |  |  |
| --- | --- | --- | --- | --- | --- | --- | --- | --- | --- | --- | --- | --- | --- | --- | --- | --- | --- | --- | --- | --- | --- | --- | --- | --- | --- | --- | --- | --- | --- | --- | --- |
|  |  | number |  | PDM/HMM | | | |  |  |  |  |  |  |  |  |  |  | GCV/SSC | | | |  |  |  |  |  |  |  |  |  |  |
|  |  |  |  | 1 | 2 | 3 | 4 | 5 | 6 | 7 | 8 | 9 | 10 | 11 | 12 | 13 | 14 | 1 | 2 | 3 | 4 | 5 | 6 | 7 | 8 | 9 | 10 | 11 | 12 | 13 | 14 |
|  |  |  |  |  |  |  |  |  |  |  |  |  |  |  |  |  |  |  |  |  |  |  |  |  |  |  |  |  |  |  |  |
| 3 | α and β-tubulin | 9 | 0.7251 |  |  |  |  |  |  |  |  |  | **∙** | **∙** | **∙** | **∙** | **∙** |  |  |  |  |  |  |  |  |  | **∙** | **∙** | **∙** | **∙** | **∙** |
| 4 | histone H3 | 7 | 0.6451 |  |  |  |  |  |  |  | **∙** | **∙** | **∙** | **∙** | **∙** | **∙** | **∙** |  |  |  |  |  |  |  | **∙** | **∙** | **∙** | **∙** | **∙** | **∙** | **∙** |
| 6 | Tb927.1.4540 | 6* | 0.5549 |  |  |  |  |  |  | **∙** | **∙** | **∙** | **∙** | **∙** | **∙** | **∙** | **∙** |  |  |  |  |  |  | **∙** | **∙** | **∙** | **∙** | **∙** | **∙** | **∙** | **∙** |
| 9 | 65 kDa invariant surface glycoprotein | 6* | 0.4533 | X |  |  |  |  | X | **∙** | **∙** | **∙** | **∙** | **∙** | **∙** | **∙** | **∙** | X |  |  | X |  | X | **∙** | **∙** | **∙** | **∙** | **∙** | **∙** | **∙** | **∙** |
| 12 | Tb927.2.5290 | 8 | 0.3635 |  |  |  |  |  |  |  |  | **∙** | **∙** | **∙** | **∙** | **∙** | **∙** | X |  |  |  |  | X | X |  | **∙** | **∙** | **∙** | **∙** | **∙** | **∙** |
| 13 | Tb927.3.2550 | 5 | 0.4167 |  |  |  |  |  | **∙** | **∙** | **∙** | **∙** | **∙** | **∙** | **∙** | **∙** | **∙** |  |  |  |  |  | **∙** | **∙** | **∙** | **∙** | **∙** | **∙** | **∙** | **∙** | **∙** |
| 17 | Tb927.3.4070 | 5 | 0.5029 | X | X | X | X | X | **∙** | **∙** | **∙** | **∙** | **∙** | **∙** | **∙** | **∙** | **∙** | X |  | X |  | X | **∙** | **∙** | **∙** | **∙** | **∙** | **∙** | **∙** | **∙** | **∙** |
| 18 | 73 kDa paraflagellar rod protein | 5 | 0.7398 |  |  |  |  |  | **∙** | **∙** | **∙** | **∙** | **∙** | **∙** | **∙** | **∙** | **∙** |  |  |  |  |  | **∙** | **∙** | **∙** | **∙** | **∙** | **∙** | **∙** | **∙** | **∙** |
| 20 | Tb927.3.5690 | 5 | 0.3687 |  |  |  |  |  | **∙** | **∙** | **∙** | **∙** | **∙** | **∙** | **∙** | **∙** | **∙** |  |  |  |  |  | **∙** | **∙** | **∙** | **∙** | **∙** | **∙** | **∙** | **∙** | **∙** |
| 24 | serine/threonine-protein phosphatase PP1 | 5 | 0.435 |  |  |  |  | **∙** | **∙** | **∙** | **∙** | **∙** | **∙** | **∙** | **∙** | **∙** | **∙** |  |  |  |  | **∙** | **∙** | **∙** | **∙** | **∙** | **∙** | **∙** | **∙** | **∙** | **∙** |
| 26 | amino acid transporter | 4 | 0.5009 |  |  |  |  | **∙** | **∙** | **∙** | **∙** | **∙** | **∙** | **∙** | **∙** | **∙** | **∙** |  |  |  |  | **∙** | **∙** | **∙** | **∙** | **∙** | **∙** | **∙** | **∙** | **∙** | **∙** |
| 28 | receptor-type adenylate cyclase GRESAG 4 | 7 | 0.3709 | X | X |  | X |  |  | X | **∙** | **∙** | **∙** | **∙** | **∙** | **∙** | **∙** | X | X | X | X | X | X | X | **∙** | **∙** | **∙** | **∙** | **∙** | **∙** | **∙** |
| 29 | amino acid transporter 10 | 6 | 0.4674 | X | X |  |  |  |  | **∙** | **∙** | **∙** | **∙** | **∙** | **∙** | **∙** | **∙** | X | X |  |  |  |  | **∙** | **∙** | **∙** | **∙** | **∙** | **∙** | **∙** | **∙** |
| 30 | UDP-GlcNAc-dependent glycosyltransferase | 7 | 0.3885 |  |  |  |  |  |  |  | **∙** | **∙** | **∙** | **∙** | **∙** | **∙** | **∙** |  |  |  |  |  |  |  | **∙** | **∙** | **∙** | **∙** | **∙** | **∙** | **∙** |
| 32 | 75 kDa invariant surface glycoprotein | 4 | 0.492 |  |  |  |  | **∙** | **∙** | **∙** | **∙** | **∙** | **∙** | **∙** | **∙** | **∙** | **∙** |  |  | X | X | **∙** | **∙** | **∙** | **∙** | **∙** | **∙** | **∙** | **∙** | **∙** | **∙** |
| 39 | histone H4 | 10 | 0.6173 |  |  |  |  |  |  |  |  |  |  | **∙** | **∙** | **∙** | **∙** |  |  |  |  |  |  |  |  |  |  | **∙** | **∙** | **∙** | **∙** |
| 42 | receptor-type adenylate cyclase GRESAG 4 | 5 | 0.4895 |  |  |  |  |  | **∙** | **∙** | **∙** | **∙** | **∙** | **∙** | **∙** | **∙** | **∙** | X |  |  |  | X | **∙** | **∙** | **∙** | **∙** | **∙** | **∙** | **∙** | **∙** | **∙** |
| 43 | cysteine peptidase | 11 | 0.574 |  |  |  |  |  |  |  |  |  |  |  | **∙** | **∙** | **∙** |  |  |  |  |  |  |  |  |  |  |  | **∙** | **∙** | **∙** |
| 44 | Tb927.6.1300 | 5* | 0.3389 |  | X | X | X | X | **∙** | **∙** | **∙** | **∙** | **∙** | **∙** | **∙** | **∙** | **∙** |  | X | X | X | X | **∙** | **∙** | **∙** | **∙** | **∙** | **∙** | **∙** | **∙** | **∙** |
| 47 | S-adenosylmethionine synthetase | 9 | 0.5884 |  |  |  |  |  |  |  |  |  | **∙** | **∙** | **∙** | **∙** | **∙** |  |  |  |  |  |  |  |  |  | **∙** | **∙** | **∙** | **∙** | **∙** |
| 55 | retrotransposon hot spot protein 7 (RHS7) | 9 | 0.3543 | X |  |  |  | X |  | X | X |  | **∙** | **∙** | **∙** | **∙** | **∙** | X | X | X | X | X |  | X | X |  | **∙** | **∙** | **∙** | **∙** | **∙** |
| 57 | histone H2A | 13 | 0.7233 |  |  |  |  |  |  |  |  |  |  |  |  |  | **∙** |  |  |  |  |  |  |  |  |  |  |  |  |  | **∙** |
| 61 | Tb927.7.5930 | 8 | 0.512 |  | X | X |  |  |  |  |  | **∙** | **∙** | **∙** | **∙** | **∙** | **∙** | X | X | X | X | X |  | X | X | **∙** | **∙** | **∙** | **∙** | **∙** | **∙** |
| 62 | receptor-type adenylate cyclase GRESAG 4 | 5 | 0.4521 | X | X | X | X | X | **∙** | **∙** | **∙** | **∙** | **∙** | **∙** | **∙** | **∙** | **∙** | X | X |  | X | X | **∙** | **∙** | **∙** | **∙** | **∙** | **∙** | **∙** | **∙** | **∙** |
| 62a | Tb07.2F2.380 | 4* | 0.4477 | X | X | X | X | **∙** | **∙** | **∙** | **∙** | **∙** | **∙** | **∙** | **∙** | **∙** | **∙** | X | X |  | X | **∙** | **∙** | **∙** | **∙** | **∙** | **∙** | **∙** | **∙** | **∙** | **∙** |
| 64a | nucleolar RNA-binding protein | 4 | 0.3072 |  |  |  |  | **∙** | **∙** | **∙** | **∙** | **∙** | **∙** | **∙** | **∙** | **∙** | **∙** |  |  |  |  | **∙** | **∙** | **∙** | **∙** | **∙** | **∙** | **∙** | **∙** | **∙** | **∙** |
| 67 | major surface protease gp63 | 4 | 0.474 |  |  |  |  | **∙** | **∙** | **∙** | **∙** | **∙** | **∙** | **∙** | **∙** | **∙** | **∙** |  |  |  |  | **∙** | **∙** | **∙** | **∙** | **∙** | **∙** | **∙** | **∙** | **∙** | **∙** |
| 72 | amino acid transporter | 5 | 0.5149 |  |  |  |  |  | **∙** | **∙** | **∙** | **∙** | **∙** | **∙** | **∙** | **∙** | **∙** |  |  |  |  |  | **∙** | **∙** | **∙** | **∙** | **∙** | **∙** | **∙** | **∙** | **∙** |
| 73 | PFR2 69 kDa paraflagellar rod protein | 5 | 0.6863 |  |  |  |  |  | **∙** | **∙** | **∙** | **∙** | **∙** | **∙** | **∙** | **∙** | **∙** |  |  |  |  |  | **∙** | **∙** | **∙** | **∙** | **∙** | **∙** | **∙** | **∙** | **∙** |
| 75 | Tb927.8.6700 | 4 | 0.42 | X | X | X |  | **∙** | **∙** | **∙** | **∙** | **∙** | **∙** | **∙** | **∙** | **∙** | **∙** | X | X | X |  | **∙** | **∙** | **∙** | **∙** | **∙** | **∙** | **∙** | **∙** | **∙** | **∙** |
| 80 | amino acid transporter | 11 | 0.3786 | X | X |  |  |  |  | X |  |  | X |  | **∙** | **∙** | **∙** | X |  | X | X | X | X | X | X | X |  |  | **∙** | **∙** | **∙** |
| 80a | receptor-type adenylate cyclase GRESAG 4 | 8 | 0.3811 |  |  |  | X | X | X |  | X | **∙** | **∙** | **∙** | **∙** | **∙** | **∙** | X | X |  | X | X |  |  |  | **∙** | **∙** | **∙** | **∙** | **∙** | **∙** |
| 82 | fatty acyl CoA syntetase | 4 | 0.5232 | X | X | X |  | **∙** | **∙** | **∙** | **∙** | **∙** | **∙** | **∙** | **∙** | **∙** | **∙** | X |  | X |  | **∙** | **∙** | **∙** | **∙** | **∙** | **∙** | **∙** | **∙** | **∙** | **∙** |
| 85 | Tb09.v1.0470 | 5* | 0.3717 |  | X | X | X | X | **∙** | **∙** | **∙** | **∙** | **∙** | **∙** | **∙** | **∙** | **∙** |  | X | X | X | X | **∙** | **∙** | **∙** | **∙** | **∙** | **∙** | **∙** | **∙** | **∙** |
| 87a | Tb09.211.1000 | 4 | 0.3728 |  |  |  |  | **∙** | **∙** | **∙** | **∙** | **∙** | **∙** | **∙** | **∙** | **∙** | **∙** |  |  |  |  | **∙** | **∙** | **∙** | **∙** | **∙** | **∙** | **∙** | **∙** | **∙** | **∙** |
| 89 | glycerol kinase | 5 | 0.6443 |  |  |  |  |  | **∙** | **∙** | **∙** | **∙** | **∙** | **∙** | **∙** | **∙** | **∙** |  |  |  |  |  | **∙** | **∙** | **∙** | **∙** | **∙** | **∙** | **∙** | **∙** | **∙** |
| 90 | ADP-ribosylation factor | 4 | 0.5368 |  |  |  |  | **∙** | **∙** | **∙** | **∙** | **∙** | **∙** | **∙** | **∙** | **∙** | **∙** |  |  |  |  | **∙** | **∙** | **∙** | **∙** | **∙** | **∙** | **∙** | **∙** | **∙** | **∙** |
| 93 | BARP protein | 14 | 0.3083 | X | X | X | X | X | X | X | X | X | X | X | X | X | X |  | X |  | X | X | X | X | X | X |  | X |  | X |  |
| 103 | Tb10.70.0040 | 5 | 0.3771 |  | X | X |  |  | **∙** | **∙** | **∙** | **∙** | **∙** | **∙** | **∙** | **∙** | **∙** |  | X | X |  |  | **∙** | **∙** | **∙** | **∙** | **∙** | **∙** | **∙** | **∙** | **∙** |
| 105a | expression site-associated gene (ESAG) protein | 4 | 0.4197 |  |  | * |  | **∙** | **∙** | **∙** | **∙** | **∙** | **∙** | **∙** | **∙** | **∙** | **∙** |  |  | * |  | **∙** | **∙** | **∙** | **∙** | **∙** | **∙** | **∙** | **∙** | **∙** | **∙** |
| 106 | procyclin-associated gene | 4 | 0.5278 |  |  |  |  | **∙** | **∙** | **∙** | **∙** | **∙** | **∙** | **∙** | **∙** | **∙** | **∙** |  |  |  |  | **∙** | **∙** | **∙** | **∙** | **∙** | **∙** | **∙** | **∙** | **∙** | **∙** |
| 107 | histone H2B | 14 | 0.7885 |  |  |  |  |  |  |  |  |  |  |  |  |  |  |  |  |  |  |  |  |  |  |  |  |  |  |  |  |
| 109c | Tb10.389.0830 | 4 | 0.3182 |  | X |  | X | **∙** | **∙** | **∙** | **∙** | **∙** | **∙** | **∙** | **∙** | **∙** | **∙** |  | X |  | X | **∙** | **∙** | **∙** | **∙** | **∙** | **∙** | **∙** | **∙** | **∙** | **∙** |
| 112 | DNA polymerase kappa | 10 | 0.3715 | X | X | X | X | X | X |  |  | X | X | **∙** | **∙** | **∙** | **∙** |  |  |  | X | X | X |  | X | X | X | **∙** | **∙** | **∙** | **∙** |
| 113 | cation transporter | 5 | 0.4712 |  |  |  |  |  | **∙** | **∙** | **∙** | **∙** | **∙** | **∙** | **∙** | **∙** | **∙** |  |  |  |  |  | **∙** | **∙** | **∙** | **∙** | **∙** | **∙** | **∙** | **∙** | **∙** |
| 117 | calmodulin | 4 | 0.6032 |  |  |  |  | **∙** | **∙** | **∙** | **∙** | **∙** | **∙** | **∙** | **∙** | **∙** | **∙** |  |  |  |  | **∙** | **∙** | **∙** | **∙** | **∙** | **∙** | **∙** | **∙** | **∙** | **∙** |
| x1 | ribonucleoside-diphosphate reductase small chain | 4 | 0.5809 |  |  |  |  | **∙** | **∙** | **∙** | **∙** | **∙** | **∙** | **∙** | **∙** | **∙** | **∙** |  |  |  |  | **∙** | **∙** | **∙** | **∙** | **∙** | **∙** | **∙** | **∙** | **∙** | **∙** |
|  |  |  |  |  |  |  |  |  |  |  |  |  |  |  |  |  |  |  |  |  |  |  |  |  |  |  |  |  |  |  |  |

NB: the number of gene duplicates in a tandem array is represented by open squares. Gene duplicates implicated in gene conversion events are marked with a cross X, those unaffected are left blank. An asterisk * denotes an ectopic gene conversion event.
