# Supplementary material for: Tandem gene arrays in Trypanosoma brucei: Comparative phylogenomic analysis of duplicate sequence variation
Source: BMC Evol Biol. 2007 Apr 4;7:54. doi: 10.1186/1471-2148-7-54 (PMC1855330; doi:10.1186/1471-2148-7-54)
Supplement: Additional File 7 — Table S2. Gene conversion events catalogued by sequence individual gene duplicate sequence [file 1471-2148-7-54-S7.doc]

**Table S2. Gene conversion events by sequence**

| Identifier | Chr | Description | Recombinant region: | | | Location: | p-value |  | Donor | Recipient | Voucher sequences: | |  |
| --- | --- | --- | --- | --- | --- | --- | --- | --- | --- | --- | --- | --- | --- |
|  |  |  | Length | Start | Finish |  | GCV | SSC |  |  | 1 | 2 | 3 |
|  |  |  |  |  |  |  |  |  |  |  |  |  |  |
|  |  |  |  |  |  |  |  |  |  |  |  |  |  |
| 9 | 2 | 65 kDa invariant surface glycoprotein | 708 | 1243 | 1951 | 5'UTR | 3.66E-10 | 2.38E-07 | 6 | 4 |  |  |  |
|  |  |  | 114 | 1969 | 2083 | CDS | 4.66E-08 | 2.24E-10 | 6 | 1 |  |  |  |
|  |  |  | 643 | 1966 | 2609 | CDS | 9.24E-11 | 1.11E-12 | 6 | 1 |  |  |  |
| 12 | 2 | Tb927.2.5290 | 297 | 605 | 902 | CDS | 3.64E-07 | 4.02E-02 | 6 or 7 | 1 |  |  |  |
| 17 | 3 | Tb927.3.4070 | 34 | 5 | 39 | 5'UTR | 1.33E-13 | 9.87E-07 | 3 and 5 | 3 and 5 |  |  |  |
|  |  |  | 47 | 1737 | 1784 | CDS | 8.12E-03 | 4.71E-05 | 3 and 5 | 3 and 5 |  |  |  |
|  |  |  | 206 | 2057 | 2263 | 3'UTR | 4.62E-03 | 4.88E-21 | 5 or 1 | 3 |  |  |  |
| 28 | 4 | receptor-type adenylate cyclase GRESAG 4 | 504 | 281 | 785 | CDS | 1.76E-15 | 1.61E-14 | 1 | 6 |  |  |  |
|  |  |  | 35 | 977 | 1012 | CDS | 6.33E-09 | 3.21E-04 | 6 | 2 |  |  |  |
|  |  |  | 125 | 897 | 1022 | CDS | 1.16E-13 | 6.31E-12 | 1 | 4 |  |  |  |
|  |  |  | 303 | 980 | 1283 | CDS | 1.20E-09 | 5.57E-10 | 7 | 5 |  |  |  |
|  |  |  | 525 | 1306 | 1831 | CDS | 1.01E-15 | 7.80E-12 | 4 | 2 |  |  |  |
|  |  |  | 324 | 1828 | 2152 | CDS | 3.25E-16 | 2.14E-04 | 3 | 1 |  |  |  |
| 29 | 4 | amino acid transporter 10 | 25 | 511 | 536 | CDS | 6.07E-06 | 5.78E-05 | 1, 3 or 5 | 2 |  |  |  |
| 32 |  | 75 kDa invariant surface glycoprotein | 28 | 136 | 164 | CDS | 9.61E-04 | 3.97E-02 | T* | T* |  |  |  |
|  |  |  | 195 | 408 | 603 | CDS | 1.21E-11 | 5.48E-11 | 2 | T |  |  |  |
|  |  |  | 59 | 1237 | 1296 | CDS | 9.66E-04 | 2.21E-05 | 4 | 3 |  |  |  |
| 42 | 6 | receptor-type adenylate cyclase GRESAG 4 | 261 | 1370 | 1631 | CDS | 1.88E-07 | ns | 1 or 4 | 5 |  |  |  |
| 44 | 6 | Tb927.6.1300 | 102 | 1551 | 1653 | 5'UTR | 3.62E-04 | 6.61E-07 | 3 and 4 | 3 and 4 |  |  |  |
|  |  |  | 172 | 2625 | 2797 | CDS | 1.91E-12 | 1.13E-14 | 3 | 4 |  |  |  |
|  |  |  | 190 | 3699 | 3889 | 3'UTR | 1.29E-14 | 9.21E-10 | 2 and 5 | 2 and 5 |  |  |  |
| 55 | 7 | retrotransposon hot spot protein 7 (RHS7) | 146 | 1556 | 1702 | CDS | 1.42E-03 | 6.27E-02 | 5 | 8 |  |  |  |
|  |  |  | 84 | 1689 | 1773 | CDS | 8.82E-03 | 3.72E-06 | 5 | 1 |  |  |  |
|  |  |  | 134 | 1708 | 1842 | CDS | 1.61E-04 | 3.28E-03 | 3 | 1 |  |  |  |
|  |  |  | 98 | 1850 | 1948 | CDS | 1.87E-03 | 1.45E-03 | 7 | 4 |  |  |  |
|  |  |  | 769 | 3781 | 4550 | CDS | 3.20E-08 | 1.40E-05 | 8 | 2 |  |  |  |
| 61 | 7 | Tb927.7.5930 | 165 | 381 | 546 | CDS | 6.50E-06 | 2.14E-08 | 1 | 8 |  |  |  |
|  |  |  | 110 | 625 | 735 | CDS | 5.77E-11 | 1.83E-07 | 4 | 3 |  |  |  |
|  |  |  | 89 | 700 | 789 | CDS | 2.82E-12 | 2.41E-07 | 3 | 2 |  |  |  |
|  |  |  | 105 | 937 | 1042 | CDS | 1.14E-12 | 2.14E-09 | 4 | 2 |  |  |  |
|  |  |  | 52 | 1085 | 1137 | CDS | 2.65E-13 | 1.33E-05 | 1 | 8 |  |  |  |
|  |  |  | 97 | 1205 | 1302 | CDS | 3.89E-02 | 2.83E-06 | 5 or 7 | 8 |  |  |  |
|  |  |  | 71 | 1408 | 1479 | CDS | 3.95E-03 | 8.31E-05 | 8 | 2 |  |  |  |
|  |  |  | 84 | 1393 | 1477 | CDS | 7.34E-12 | 3.41E-05 | 8 | 1 |  |  |  |
|  |  |  | 300 | 1391 | 1691 | CDS | 1.92E-09 | 5.61E-04 | 1 | 8 |  |  |  |
|  |  |  | 21 | 1626 | 1647 | CDS | 6.50E-04 | 4.03E-05 | 2 | 8 |  |  |  |
|  |  |  | 166 | 1648 | 1814 | CDS | 9.97E-16 | 4.25E-12 | 5 or 7 | 2 |  |  |  |
| 62 | 7 | receptor-type adenylate cyclase GRESAG 4 | 330 | 120 | 450 | 5'UTR-CDS | 1.13E-15 | 4.74E-10 | 5 | 4 |  |  |  |
|  |  |  | 373 | 3632 | 4005 | CDS-3'UTR | 2.06E-15 | 3.38E-15 | 5 | 2 |  |  |  |
|  |  |  | 314 | 4086 | 4400 | 3'UTR | 1.27E-14 | 3.16E-09 | 5 or 1 | 2 |  |  |  |
| 62a | 7 | Tb927.7.6110 | 56 | 1 | 57 | CDS | 7.78E-16 | 3.44E-10 | 1 and 2 | 1 and 2 |  |  |  |
|  |  |  | 30 | 101 | 131 | CDS | 3.57E-04 | ns | 1 and 4 | 1 and 4 |  |  |  |
| 75 | 8 | Tb927.8.6700 | 71 | 318 | 389 | CDS | 4.83E-11 | 4.75E-12 | 1 | 3 |  |  |  |
|  |  |  | 54 | 803 | 857 | CDS | 4.47E-09 | 3.53E-03 | 1 | 2 |  |  |  |
|  |  |  | 91 | 1098 | 1189 | CDS | ns | 1.11E-04 | 1 | 3 |  |  |  |
|  |  |  | 145 | 2058 | 2203 | 3'UTR | 1.43E-13 | 8.99E-07 | 1 | 2 |  |  |  |
|  |  |  | 355 | 2204 | 2559 | 3'UTR | 7.06E-13 | 1.08E-07 | 1 | 3 |  |  |  |
| 80 | 8 | amino acid transporter | 48 | 1 | 49 | CDS | 1.02E-03 | 3.42E-05 | 8 | 5 |  |  |  |
|  |  |  | 205 | 1 | 206 | CDS | 1.50E-17 | 2.08E-06 | 3 | 1 |  |  |  |
|  |  |  | 81 | 548 | 629 | CDS | 6.95E-03 | 2.56E-05 | 4 | 1 |  |  |  |
|  |  |  | 19 | 1042 | 1061 | CDS | 2.59E-03 | 3.83E-03 | 9 | 1 |  |  |  |
|  |  |  | 151 | 1086 | 1237 | CDS | 1.03E-05 | 2.44E-03 | 6 | 7 |  |  |  |
| 80a | 8 | receptor-type adenylate cyclase GRESAG 4 | 315 | 1665 | 1980 | CDS | 1.38E-04 | 1.60E-04 | 1 or 2 | 5 |  |  |  |
|  |  |  | 58 | 5028 | 5086 | 3'UTR | 5.59E-03 | 7.86E-03 | 1 or 2 | 4 |  |  |  |
| 85 | 9 | Tb09.v1.0470 | 73 | 294 | 367 | CDS | 4.30E-07 | 2.76E-02 | 4 | 3 | TP2H7-1b07.p1ka | tryp_IXb-365f11.p1c | TP28A6-1e11.p1k |
|  |  |  | 214 | 812 | 1026 | CDS | 2.35E-13 | 1.94E-03 | 5 | 3 | tryp_IXb-283f11.q1c |  | TP2H7-1d11.q1k |
|  |  |  | 161 | 1767 | 1928 | CDS | 5.71E-04 | 2.20E-04 | 2 | 5 | TP2H7-1d04.p1k | TP2H7-1b07.q1k | TP25J20-1e06.p1k |
|  |  |  | 150 | 3569 | 3719 | CDS | 1.89E-06 | 7.00E-09 | 5 and 4 | 5 and 4 | tryp_IXb-248e11.p1c | tryp_IXb-106a12.p1c | tryp_IXb-391f10.p1c |
|  |  |  | 154 | 5712 | 5866 | CDS | 1.27E-12 | 1.32E-04 | 5 | 3 | tryp_IXb-274g07.q1c |  | tryp_IXb-146c02.p1c |
| 93 | 9 | BARP protein | 110 | 21 | 131 | 5'UTR | 1.39E-02 | 6.35E-03 | 7 | 2 | tryp_IXb-11g04.q1c | tryp_IXb-257a06.q1c | tryp_IXa-25f10.q1c |
|  |  |  | 237 | 93 | 330 | CDS | 1.99E-12 | 1.04E-03 | 6 | 2 | tryp_IXb-367b11.p1c | tryp_IXb-11g04.q1c | tryp_IXa-34g03.p1k |
|  |  |  | 55 | 209 | 264 | CDS | 4.09E-08 | 2.01E-08 | 4 or 7 | 14 | tryp_IXb-190b11.p1k | tryp_IXb-294f07.q1c | tryp_IXb-175e10.q2k4187 |
|  |  |  | 20 | 745 | 765 | CDS | 1.64E-05 | 1.80E-03 | 14 | 13 | tryp_IXb-375b09.p1k | tryp_IXb-29c08.q1c | tryp_IXb-379f06.p1k |
|  |  |  | 335 | 865 | 1200 | CDS-3'UTR | 4.96E-13 | 7.88E-11 | 6 | 4 or 7 | tryp_IXb-337c02.p1c | tryp_IXb-214h12.q1c | tryp_IXb-368e11.q1k |
|  |  |  | 358 | 995 | 1353 | 3'UTR | 1.54E-10 | 9.31E-11 | 8 | 7 | tryp_IXb-368e11.q1k | tryp_IXb-227g11.p1c | tryp_IXb-228h01.q1c |
|  |  |  | 391 | 1190 | 1581 | 3'UTR | 2.79E-03 | 2.00E-08 | 2 | 5 | tryp_IXb-276b11.q1c | tryp_IXb-104a11.p1c | tryp_IXb-236g02.p1c |
|  |  |  | 170 | 1310 | 1480 | 3'UTR | 1.18E-15 | 1.35E-13 | 9 | 7 | tryp_IXb-157b01.p1c | tryp_IXb-368b11.p1c | tryp_IXb-375b09.q1c |
|  |  |  | 308 | 1409 | 1717 | 3'UTR | 8.43E-09 | 2.74E-06 | 11 | 8 | tryp_IXb-31e12.q1c | tryp_IXb-213c08.p1c | tryp_IXb-368b11.p1c |
| 103 | 10 | Tb10.70.0040 | 165 | 157 | 322 | 5'UTR | 2.73E-04 | 6.70E-03 | 3 | 2 | tryp_X-350e08.q1c | tryp_X-187f11.p1c | tryp_X-169h03.p1c |
| 105a | 10 | expression site-associated gene (ESAG) protein | 109 | 519 | 628 | CDS | ns | 9.88E-04 | T | 3 | tryp_X-241a04.p1c | tryp_X-290b08.p1c | tryp_X-235f03.q1c |
| 109c | 10 | Tb10.389.0830 | 22 | 223 | 245 | CDS | 2.48E-05 | 1.97E-06 | 2 | 4 | tryp_X-62h07.p1c | tryp_X-234h03.q1c | tryp_X-82a03.q1c |
| 112 | 11 | DNA polymerase kappa | 310 | 315 | 625 | 5'UTR-CDS | 1.92E-11 | 3.66E-11 | 4 or 5 | 6 |  |  |  |
|  |  |  | 104 | 820 | 924 | CDS | 1.85E-11 | 1.65E-17 | 10 | 8 | tryp_XI-974d08.q1k | tryp_XI-878f11.p1k |  |
|  |  |  | 246 | 893 | 1139 | CDS | 1.66E-09 | 5.01E-21 | 9 or 8 | 8 or 9 | tryp_XI-991e11.q1k | tryp_XI-891a07.q1k | tryp_XI-340c09.p1k |
|  |  |  | 78 | 1617 | 1695 | CDS | 3.19E-02 | 9.62E-04 | 9 | 6 | tryp_XI-974d08.p1k | tryp_XI-950d11.p1k | tryp_XI-341d09.q1ka |
|  |  |  | 56 | 1751 | 1807 | CDS | 6.36E-02 | 5.04E-04 | 3 or 9 | 6 | tryp_XI-974d08.p1k | tryp_XI-336e01.q1ka | tryp_XI-929d06.p1k |
|  |  |  |  |  |  |  |  |  |  |  |  |  |  |
