# Supplementary material for: Tandem gene arrays in Trypanosoma brucei: Comparative phylogenomic analysis of duplicate sequence variation
Source: BMC Evol Biol. 2007 Apr 4;7:54. doi: 10.1186/1471-2148-7-54 (PMC1855330; doi:10.1186/1471-2148-7-54)
Supplement: Additional File 9 — Table S3. Identity and position of tandem gene pairs and arrays in T. brucei. [file 1471-2148-7-54-S9.doc]

**Table S3. Identity and position of tandem gene pairs and arrays in *T. brucei*.**

| Array | Gene name or GeneDB identifer | Chr. | Repeat | Size (bp) | Position: |  | SP* | TMH^#^ | Pfam domain match |
| --- | --- | --- | --- | --- | --- | --- | --- | --- | --- |
|  |  |  | number |  | Start | Finish |  |  |  |
|  |  |  |  |  |  |  |  |  |  |
| 1 | phosphoglycerate kinase | 1 | 3 | 1323-1530 | 232504 | 237299 |  |  | Phosphoglycerate kinase |
| 2 | Tb927.1.1420/1470/1500 | 1 | 3 | 1758-1992 | 360261 | 376872 |  |  |  |
| 3 | tubulin | 1 | 9 | 1329 | 570482 | 585950 |  |  |  |
| 4 | histone H3 | 1 | 7 | 402 | 592496 | 603120 |  | 13 | Core histone H2A/H2B/H3/H4 |
| 5 | pteridine transporter | 1 | 3 | 1959 | 641279 | 651238 |  |  | BT1 family |
| 5a | Tb927.1.2810-2890 | 1 | 3 | 258 | 639869 | 651577 |  |  |  |
| 5b | Tb927.1.4370-4380 | 1 | 2 | 1593-1803 | 903224 | 907602 | y | 1 |  |
| 6 | Tb927.1.4540-4650 | 1 | 6 | 1431-1582 | 939394 | 956334 | y | 3 | F-box domain |
| 7 | Tb927.1.4740-4790 | 1 | 2 | 3087-3180 | 971957 | 979487 |  |  |  |
| 8 | Tb927.2.2520 | 2 | 2 | 813 | 494285 | 496413 |  |  | Bacterial transferase hexapeptide repeat |
| 9 | 65 kDa invariant surface glycoprotein | 2 | 6 | 1311 | 598050 | 617079 |  | 1 |  |
| 10 | cysteine peptidase | 2 | 2 | 843 | 631650 | 634491 | y | 1 | CHAP domain |
| 11 | branched-chain amino acid aminotransferase | 2 | 2 | 1104 | 818321 | 821263 |  |  | Aminotransferase class IV |
| 11a | kinesins | 2 | 2 | 2295/645/4569/6183 | 974913 | 1019799 |  |  | Kinesin motor domain |
| 12 | Tb927.2.5290 | 2 | 8 | 1104-1110 | 942660 | 957295 |  | 2 | Mucin-like glycoprotein |
| 12a | Tb03.48O8.550-540 | 3 | 2 | 321 | 603856 | 605085 | y | 1 |  |
| 12b | Tb03.1J15.580-90 | 3 | 2 | 3762 | 323362 | 330879 |  |  | Keratin, high sulfur B2 protein/ankyrin |
| 13 | Tb03.48O8.300 | 3 | 5 | 657-918 | 640907 | 647949 | y |  |  |
| 14 | 60S ribosomal protein L13 | 3 | 2 | 690 | 850898 | 852457 |  |  | Ribosomal protein L13e |
| 15 | kinesin | 3 | 2 | 1776 | 866616 | 870403 |  |  | Kinesin motor domain |
| 16 | lipase domain protein | 3 | 2 | 3849 | 1071344 | 1079769 |  | 6 | Lipase (class 3) |
| 16a | tryparedoxin/Tb03.28C22.730 | 3 | 2 | 435/861 | 1050464 | 1052403 |  |  | Thioredoxin/Calcium-binding EF-hand |
| 17 | Tb03.26J7.120 | 3 | 5 | 1758-1788 | 1148265 | 1159289 |  | 12 | Nodulin-like |
| 18 | 73 kDa paraflagellar rod protein | 3 | 5 | 1770 | 1208864 | 1219285 |  |  | Paraflagellar rod protein |
| 19 | dynamin/LRRP/Tb03.48K5.310/aminopeptidase | 3 | 2 | 1983/1152/1275/2616 | 1334486 | 1347912 | y |  | Dynamin central region/Leucine Rich Repeat//Peptidase family M1 |
| 20 | Tb03.2H15.520 | 3 | 5 | 993-1062 | 1592952 | 1601399 | y |  | Protein of unknown function (DUF1299) |
| 21 | ribosomal protein L3 | 4 | 2 | 1290-1443 | 448465 | 451408 |  | 1 | Ribosomal protein L3 |
| 22 | Tb04.2H8.370 | 4 | 3 | 930/1110 | 847180 | 856980 |  |  |  |
| 23 | translation elongation factor 1-beta | 4 | 2 | 786/312 | 915105 | 918334 |  |  | EF-1 guanine nucleotide exchange domain |
| 24 | serine/threonine-protein phosphatase PP1 | 4 | 4 | 981 | 913574 | 926454 |  |  | Calcineurin-like phosphoesterase |
| 25 | receptor-type adenylate cyclase GRESAG 4, | 4 | 3 | 3699 | 1007267 | 1021293 |  | 1 | Adenylate and Guanylate cyclase catalytic domain |
| 26 | amino acid transporter | 4 | 4 | 1455 | 1059789 | 1070659 |  | 11 | Transmembrane amino acid transporter protein |
| 27 | Tb04.1D20.250 | 4 | 3 | 993/1155 | 1125479 | 1137266 |  | 1 | DM DNA binding domain/Galactosyltransferase |
| 28 | receptor-type adenylate cyclase GRESAG 4 | 4 | 7 | 3735 | 1168174 | 1200116 |  | 1 | Adenylate and Guanylate cyclase catalytic domain |
| 29 | amino acid transporter 10 | 4 | 6 | 1473 | 1319221 | 1330546 |  | 11 | Transmembrane amino acid transporter protein |
| 30 | UDP-GlcNAc-dependent glycosyltransferase | 4 | 7 | 1011-1149 | 1425330 | 1436840 |  | 1 | Galactosyltransferase |
| 31 | receptor-type adenylate cyclase GRESAG 4 | 5 | 2 | 3711 | 79857 | 88888 |  | 1 | Adenylate and Guanylate cyclase catalytic domain |
| 32 | 75 kDa invariant surface glycoprotein | 5 | 4 | 1569 | 104475 | 112561 |  | 1 | TolA protein |
| 33 | 76 kDa invariant surface glycoprotein | 5 | 2 | 1569 | 121424 | 125151 |  | 2 | TolA protein |
| 34 | Tb05.29K2.390 | 5 | 3 | 1182 | 129279 | 134297 |  | 1 | Mosquito specific cecropin |
| 35 | casein kinase I, epsilon isoform, | 5 | 2 | 993 | 269299 | 273388 |  |  | Protein kinase domain |
| 36 | oligosaccharyl transferase subunit | 5 | 3 | 2406 | 297084 | 306149 |  | 10 | Oligosaccharyl transferase STT3 subunit |
| 37 | 65 kDa invariant surface glycoprotein | 5 | 3 | 1305/525 | 448564 | 457098 |  | 1 | Apolipoprotein C-I (ApoC-1)/Leucine Rich Repeat |
| 38 | Tb05.3C6.120-270 | 5 | 3 | 405/1431/1341 | 690803 | 705230 |  | 1 | Domain of unknown function (306)/Domain of unknown function (306) |
| 39 | histone H4 | 5 | 10 | 303 | 1270989 | 1277915 |  |  | Core histone H2A/H2B/H3/H4 |
| 40 | receptor-type adenylate cyclase GRESAG 4 | 5 | 2 | 3732 | 1341202 | 1351468 |  | 1 | Adenylate and Guanylate cyclase catalytic domain |
| 41 | procyclin PARP/procyclin PARP A/GRESAG2 | 6 | 3 | 384/609/1365 | 211009 | 230660 |  | 1 | Procyclic acidic repetitive protein (PARP) |
| 42 | receptor-type adenylate cyclase GRESAG 4 | 6 | 5 | 3732 | 316098 | 338864 |  | 1 | Adenylate and Guanylate cyclase catalytic domain |
| 42a | metacaspase MCA3 | 6 | 2 | 1074 | 397321 | 423778 |  |  | Caspase domain |
| 43 | cysteine peptidase | 6 | 11 | 1353 | 404804 | 423778 |  | 1 | Papain family cysteine protease |
| 44 | Tb06.3A7.990-1190 | 6 | 5 | 576/1044 | 486546 | 504246 |  | 3 | Cyclophilin type peptidyl-prolyl cis-trans isomerase |
| 44a | polynucleotide kinase 3'-phosphatase | 6 | 2 | 1431 | 544746 | 547888 |  |  | HAD-superfamily hydrolase, subfamily IIIA |
| 45 | single strand-specific nuclease | 6 | 2 | 891 | 556535 | 559127 |  |  | S1/P1 Nuclease |
| 46 | heat shock 70 kDa protein | 6 | 2 | 1974 | 1112893 | 1117425 |  |  | Hsp70 protein |
| 47 | S-adenosylmethionine synthetase | 6 | 9 | 1194 | 1344594 | 1362628 |  |  | S-adenosylmethionine synthetase, central domain |
| 47a | 60S acidic ribosomal protein P2 | 6 | 2 | 324 | 1401849 | 1402772 |  |  | 60s Acidic ribosomal protein |
| 48 | DNA ligase | 7 | 2 | 1542 | 123400 | 127541 |  |  | ATP dependent DNA ligase domain |
| 49 | 40S ribosomal protein S16 | 7 | 2 | 450 | 268228 | 269401 |  |  | Ribosomal protein S9/S16 |
| 50 | trypanothione/tryparedoxin dependent peroxidase 1 | 7 | 3 | 501 | 290302 | 292670 |  |  | Glutathione peroxidase |
| 51 | Tb07.27M11.450-520 | 7 | 2 | 987/702 | 325419 | 332447 |  | 1 | Pentapeptide repeats (8 copies) |
| 52 | 60S ribosomal protein L7 | 7 | 3 | 729 | 429045 | 431799 |  |  | Ribosomal protein L30p/L7e |
| 53 | adenine phosphoribosyltransferase | 7 | 2 | 708 | 436819 | 438966 |  |  | Phosphoribosyl transferase domain |
| 54 | Tb07.43M14.240-330 | 7 | 2 | 288/1101/882 | 443289 | 452059 |  |  | Extensin-like protein repeat/FYVE zinc finger |
| 55 | retrotransposon hot spot protein 7 (RHS7) | 7 | 10 | 2073 | 465663 | 512454 |  |  |  |
| 56 | NADH-cytochrome b5 reductase | 7 | 2 | 864 | 705594 | 707777 |  |  | Oxidoreductase NAD-binding domain |
| 57 | histone H2A | 7 | 13 | 405 | 728982 | 740297 |  |  | Core histone H2A/H2B/H3/H4 |
| 58 | I/6 autoantigen | 7 | 2 | 741-588 | 891472 | 893109 |  |  | EF hand |
| 58a | calpain-like cysteine peptidase | 7 | 2 | 348-363 | 1075298 | 1076388 |  |  |  |
| 59 | long chain fatty acyl elongase | 7 | 3 | 906-918 | 1109482 | 1115615 |  | 7 | GNS1/SUR4 family |
| 59a | Tb07.5F10.200-170 | 7 | 3 | 792/669/636 | 1134100 | 1138174 |  | 2 | Aldehyde dehydrogenase |
| 59b | cell cycle associated protein MOB1 | 7 | 2 | 627-678 | 1501093 | 1504062 |  |  | Mob1/phocein family |
| 60 | microtubule-associated protein 1A/1B, light chain 3 | 7 | 2 | 351-363 | 1585333 | 1587331 |  |  | Microtubule associated protein 1A/1B, light chain 3 |
| 61 | Tb07.10C21.20 | 7 | 7 | 1791 | 1590223 | 1616821 |  | 13 | Transmembrane amino acid transporter protein |
| 62 | receptor-type adenylate cyclase GRESAG 4 | 7 | 5 | 3804 | 1626783 | 1657273 |  | 1 | Adenylate and Guanylate cyclase catalytic domain |
| 62a | Tb07.2F2.380 | 7 | 7 | 480-645 | 1665485 | 1670633 |  | 8 | Alkaline phytoceramidase/Cyt c heme-binding site |
| 62b | variant surface glycoprotein (VSG) | 7 | 5 | 1473 | 1768836 | 1782885 |  | 1 | Trypanosome variant surface glycoprotein |
| 63 | Tb07.15M23.130-160 | 7 | 3 | 612-708 | 1792027 | 1795431 |  | 1 | Lysis protein |
| 64 | ATP synthase F1, alpha subunit | 7 | 2 | 1755 | 2133007 | 2136851 |  |  | nucleotide-binding domain |
| 64a | nucleolar RNA-binding protein | 8 | 4 | 762/420/1101/987 | 204635 | 209907 |  |  | Nucleoplasmin |
| 64b | Tb08.12O16.630/Tb08.12O16.640 | 8 | 2 | 1413/6585 | 287095 | 305912 |  |  | Plant invertase/pectin methylesterase inhibitor/ |
| 65 | Tb08.29O4.300/vacuolar-type Ca2+-ATPase 1 | 8 | 2 | 996/3243 | 373426 | 384958 |  | 8 | H+ transporting ATPase, proton pump |
| 66 | 60S ribosomal protein L7a | 8 | 2 | 831 | 428242 | 430158 |  |  | Ribosomal protein L7Ae/L30e/S12e/Gadd45 family |
| 67 | major surface protease gp63 | 8 | 4 | 1746 | 537061 | 545385 |  | 2 | Peptidase M8, leishmanolysin metalloprotease |
| 68 | Tb08.26N11.570-590 | 8 | 2 | 600-561 | 654365 | 656584 |  |  | Sporulation related repeat |
| 69 | Tb08.26A17.40-60 | 8 | 3 | 600 | 692159 | 696084 |  |  |  |
| 70 | mannosyl-oligosaccharide 1,2-alpha-mannosidase IB | 8 | 3 | 1767 | 882717 | 888523 |  |  | Glycosyl hydrolase family 47 |
| 71 | folate transporter, | 8 | 3 | 1899 | 1085476 | 1095114 |  | 9 | Glycosyl transferase, family 4 |
| 72 | amino acid transporter | 8 | 4 | 1599-1377 | 1380123 | 1393444 |  |  |  |
| 73 | PFR2 69 kDa paraflagellar rod protein | 8 | 5 | 1803 | 1474818 | 1485236 |  |  | Paraflagellar rod protein |
| 73a | flagellar calcium-binding protein | 8 | 3 | 657/1224/702 | 1613150 | 1620711 |  |  | EF hand |
| 73b | 40S ribosomal protein S8 | 8 | 2 | 663 | 1795601 | 1797265 |  |  | Ribosomal protein S8e |
| 74 | short chain dehydrogenase/reductase | 8 | 2 | 1425-1497 | 1858415 | 1861900 |  | 1 | short chain dehydrogenase |
| 75 | Tb08.30K1.610-640 | 8 | 4 | 1785 | 1943647 | 1950764 |  | 6 |  |
| 76 | IgE-dependent histamine-releasing factor | 8 | 2 | 513 | 1953555 | 1955677 |  |  | Translationally controlled tumour protein |
| 77 | UDP-GlcNAc-dependent glycosyltransferase | 8 | 3 | 1269/1131/714 | 2057355 | 2061902 |  | 1 |  |
| 78 | Tb08.10K10.350-320 | 8 | 2 | 1086/684 | 2096002 | 2102679 |  |  | SEC-C motif |
| 79 | trans-sialidase | 8 | 2 | 2247 | 2106984 | 2111949 |  |  | BNR/Asp-box repeat |
| 80 | amino acid transporter | 8 | 10 | 1620-1641 | 2194103 | 2221475 |  |  | Transmembrane amino acid transporter protein |
| 80a | receptor-type adenylate cyclase GRESAG 4 | 8 | 8 | 3753 | 2302044 | 2340635 |  | 4 | Adenylate and Guanylate cyclase catalytic domain |
| x2 | amino acid transporter | 8 | 5 | 1383 | 2431226 | 2443741 |  | 10 | Transmembrane amino acid transporter protein |
| x3 | amino acid transporter | 8 | 2 | 1416 | 2453345 | 2458032 |  | 11 | Transmembrane amino acid transporter protein |
| 81 | 60S ribosomal protein L35, | 9 | 2 | 384 | 387861 | 388922 |  |  | Ribosomal L29 protein |
| 81a | ribosomal protein S7/Tb09.v1.0130/alpha/beta-hydrolase-like protein | 9 | 2 | 609/1086/615 | 684568 | 695774 |  |  | Isochorismatase family |
| 82 | fatty acyl CoA syntetase | 9 | 4 | 2097-2127 | 710678 | 723319 |  |  | AMP-binding enzyme |
| 83 | cAMP-specific phosphodiesterase | 9 | 2 | 2793 | 823308 | 831256 |  |  | 3'5'-cyclic nucleotide phosphodiesterase |
| 83a | arginine kinase | 9 | 3 | 1071-1215 | 946887 | 954102 |  |  | ATP:guanido phosphotransferase |
| 84 | Tb09.160.4630 | 9 | 3 | 1440 | 963128 | 968580 |  | 10 |  |
| 85 | Tb09.v1.0470 to Tb09.211.0020 | 9 | 4 | 1110/849 | 1220923 | 1240277 | y | 2 | CHCH domain |
| 85a | 60S ribosomal protein L11 | 9 | 3 | 585 | 1166701 | 1169637 |  |  | Ribosomal protein L5 |
| 86 | nascent polypeptide associated complex subunit | 9 | 2 | 567 | 1256562 | 1258096 |  |  | NAC domain |
| 87 | actin | 9 | 2 | 1131 | 1345323 | 1347967 |  |  | Actin |
| 87a | Tb09.211.1000-1030 | 9 | 4 | 819-1098 | 1417339 | 1425593 | y | 5 |  |
| 88 | 60S ribosomal subunit protein L31 | 9 | 2 | 567 | 1827516 | 1828942 |  |  | Ribosomal protein L31e |
| 88a | 60S ribosomal protein L23 | 9 | 3 | 420-438 | 1708471 | 1710166 |  |  | Ribosomal protein L14p/L23e |
| 89 | glycerol kinase | 9 | 5 | 1359 | 1888009 | 1903855 |  |  | Carbohydrate kinase, FGGY |
| 90 | ADP-ribosylation factor | 9 | 4 | 549 | 2091198 | 2094107 |  |  | ADP-ribosylation factor family |
| 91 | kinetoplastid membrane protein KMP-11 | 9 | 3 | 279 | 2097621 | 2099065 |  |  | Kinetoplastid membrane protein 11 |
| 92 | 60S ribosomal protein L5 | 9 | 2 | 927 | 2277052 | 2279256 |  |  | Ribosomal L18p/L5e family |
| 93 | BARP protein | 9 | 14 | 891 | 2338495 | 2363075 | y |  |  |
| 94 | serine carboxypeptidase (CBP1) | 10 | 3 | 1395-1401 | 228880 | 234005 | y |  | Serine carboxypeptidase |
| 95 | 40S ribosomal protein S23 | 10 | 2 | 432 | 239762 | 241005 |  |  | Ribosomal protein S12 |
| 96 | hexokinase | 10 | 2 | 1416 | 476510 | 480971 |  |  | Hexokinase |
| 97 | Elongation factor 1-alpha | 10 | 3 | 1047-1350 | 495100 | 500505 |  |  | Elongation factor Tu domain 2 |
| 97a | adenylate kinase | 10 | 2 | 669 | 607850 | 611228 |  |  | Adenylate kinase, active site lid |
| 97b | malate dehydrogenase | 10 | 2 | 957-1074 | 611503 | 613973 |  |  | Lactate/malate dehydrogenase |
| 97c | 60S acidic ribosomal protein P2 | 10 | 2 | 345 | 822736 | 823703 |  |  | 60s Acidic ribosomal protein |
| 97d | 40S ribosomal protein S3a | 10 | 2 | 771 | 992815 | 994666 |  |  | Ribosomal S3Ae family |
| 97e | Tb10.70.2850-2840 | 10 | 2 | 741 | 1084537 | 1086398 | y |  |  |
| 98 | elongation factor 2 | 10 | 2 | 2541 | 1113733 | 1119433 |  |  | Elongation factor Tu domain 2 |
| 98a | 40S ribosomal protein S18 | 10 | 2 | 462 | 1282775 | 1283955 |  |  | Ribosomal protein S13 |
| 99 | 40S ribosomal protein S10 | 10 | 2 | 660-519 | 1297940 | 1300034 |  | 1 | Plectin/S10 domain |
| 100 | procyclin-associated gene polypeptide | 10 | 2 | 1185 | 1366476 | 1368934 | y |  |  |
| 101 | Tb10.70.1290 | 10 | 2 | 696 | 1370559 | 1372516 | y |  |  |
| 102 | protein kinase | 10 | 2 | 1473 | 1436662 | 1440304 |  |  | Protein kinase domain |
| 103 | Tb10.70.0040 | 10 | 4 | 1470 | 1641891 | 1649912 |  | 11 | Protein of unknown function, DUF580 |
| 104 | 40S ribosomal protein S24E | 10 | 2 | 414 | 1778009 | 1779165 |  |  | Ribosomal protein S24e |
| 104a | eukaryotic translation initiation factor 3 subunit 8 | 10 | 2 | 2223 | 1976011 | 1981877 |  |  | translation initiation factor 3 subunit 8 |
| 105 | hexose transporter | 10 | 3 | 1584 | 2020861 | 2027401 |  | 12 | Sugar transporter |
| 105a | expression site-associated gene (ESAG) protein | 10 | 4 | 1173 | 2252001 | 2257067 | y | y |  |
| 106 | procyclin-associated gene | 10 | 4 | 1218 | 2421523 | 2426502 | y |  |  |
| 106a | procyclin | 10 | 2 | 390 | 2428196 |  | y | y | Procyclic acidic repetitive protein (PARP) |
| 107 | histone H2B | 10 | 14 | 339 | 2477495 | 2489611 |  |  | Histone core |
| 108 | RNA-binding protein | 10 | 2 | 351 | 2821941 | 2823328 |  |  | RNA-binding region RNP1 (RNA recognition motif) |
| 109 | P-type H+-ATPase | 10 | 2 | 2739 | 2907881 | 2914331 |  | 9 | E1-E2 ATPase |
| 109a | protein kinase | 10 | 2 | 828 | 3268212 | 3271203 |  |  | Protein kinase domain |
| 109b | Tb10.61.2650-2640 | 10 | 2 | 915 | 3308081 | 3310791 |  | 6 | Major intrinsic protein |
| 109c | Tb10.389.0830-0740 | 10 | 4 | 843-1425 | 2971249 | 2986804 |  |  | Zn-finger, C-x8-C-x5-C-x3-H type |
| 110 | ADP/ATP translocase 1 | 10 | 3 | 924 | 3479517 | 3483200 |  | 3 | Mitochondrial carrier protein |
| 111 | Tb10.61.1420 | 10 | 3 | 696 | 3555423 | 3558522 |  |  |  |
| 112 | DNA polymerase kappa | 11i | 10 | 1716 | 32447 | 57606 |  |  | impB/mucB/samB family |
| 113 | cation transporter | 11i | 5 | 1176 | 221689 | 233468 |  |  | ZIP Zinc transporter |
| 114 | heat shock protein 70 | 11i | 3 | 1986 | 779348 | 786243 |  |  | Hsp70 protein |
| 114a | activated protein kinase c receptor | 11i | 2 | 957 | 793759 | 796020 |  |  | WD domain, G-beta repeat |
| 115 | 40S ribosomal protein S17 | 11i | 2 | 429 | 920530 | 921715 |  |  | Ribosomal S17 |
| 116 | protein kinase | 11i | 2 | 1398 | 1063583 | 1072271 |  |  | Protein kinase domain |
| 117 | calmodulin | 11i | 4 | 450 | 1229201 | 1232167 |  |  | EF hand |
| 117a | Tb11.01.5195 | 11i | 2 | 414 | 1376824 | 1378170 |  |  | Rhodanese-like |
| 118 | mitochondrial carrier protein | 11i | 2 | 1041 | 1564064 | 1566840 |  | 2 | Mitochondrial carrier protein |
| 119 | amino acid transporter | 11i | 2 | 1389 | 1939386 | 1947371 |  | 11 | Amino acid/polyamine transporter, family II |
| 120 | 60S acidic ribosomal subunit protein | 11ii | 2 | 975 | 270839 | 273045 |  |  | Ribosomal protein L10 |
| 121 | 40s ribosomal protein S4 | 11ii | 2 | 822 | 735669 | 737560 |  |  | KOW motif |
| 122 | nucleobase transporter | 11ii | 2 | 1308 | 737853 | 742138 | y | 9 | Nucleoside transporter |
| 122a | 60S ribosomal protein L28 | 11ii | 2 | 441 | 1411928 | 1413935 |  |  | Ribosomal L28e protein |
| 123 | malic enzyme | 11ii | 2 | 1695 | 1216372 | 1221551 |  |  | Malic oxidoreductase |
| 124 | 40S ribosomal protein S21 | 11ii | 2 | 585 | 1503149 | 1504595 |  |  | Ribosomal protein S21e |
| 125 | pif1 helicase-like protein | 11ii | 2 | 2790 | 1608535 | 1615539 | y |  | AAA ATPase |
| 125a | Tb11.02.5480-5490 | 11ii | 2 | 390 | 1793041 | 1794616 | y | 4 | Protein of unknown function (DUF423) |
| 126 | Gp63-1 surface protease homolog | 11ii | 3 | 1686 | 1830171 | 1834766 | y |  | Leishmanolysin |

* The presence of a signal peptide is denoted by a ‘y’.

# The number of trans-membrane helices.
